# Highly Stereoselective (3+2) Cycloadditions of Levoglucosenone (LGO) with the In Situ-Generated Thiocarbonyl *S*-Methanides (Thiocarbonyl Ylides) Derived from Aromatic and Cycloaliphatic Thioketones †

**DOI:** 10.3390/molecules31132198

**Published:** 2026-06-23

**Authors:** Grzegorz Mlostoń, Małgorzata Celeda, Marcin Palusiak, Heinz Heimgartner, Zbigniew J. Witczak

**Affiliations:** 1 Department of Organic and Applied Chemistry, Faculty of Chemistry, University of Lodz, Tamka 12, 91-403 Lodz, Poland; malgorzata.celeda@chemia.uni.lodz.pl; 2 Department of Physical Chemistry, Faculty of Chemistry, University of Lodz, Pomorska 163/165, 90-236 Lodz, Poland; marcin.palusiak@chemia.uni.lodz.pl; 3 Department of Chemistry, University of Zurich, Winterthurerstrasse 190, CH-8057 Zurich, Switzerland; heinz.heimgartner@chem.uzh.ch; 4 Department of Pharmaceutical Sciences, Nesbitt School of Pharmacy, Wilkes University, 84 W. South Street, Wilkes-Barre, PA 18766, USA; zbigniew.witczak@wilkes.edu

**Keywords:** levoglucosenone, thiocarbonyl *S*-methanides, (3+2) cycloadditions, sulfur heterocycles, single-crystal X-ray analysis

## Abstract

The in situ-generated thiocarbonyl S-methanides derived from cycloaliphatic thioketones undergo (3+2) cycloaddition onto the C=C bond of levoglucosenone yielding anticipated, polycyclic tetrahydrothiophene derivatives in a regio- and stereoselective manner. The cycloaddition process occurred stereoselectively via the less hindered exo-face approach; exo-diastereoisomers were formed in all studied reactions. Some of the obtained crystalline (3+2) cycloadducts were studied by the monocrystal X-ray diffraction analysis, which unambiguously confirmed the postulated structure. Stable (3+2) cycloadducts were isolated in good yields (50–80%).

## 1. Introduction

**Figure 1:**
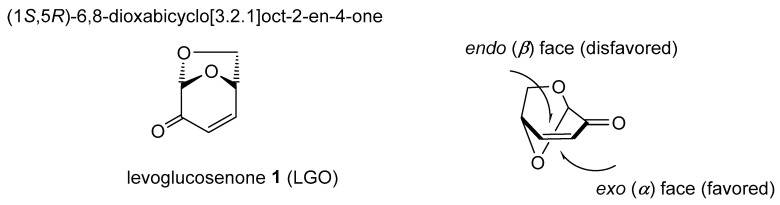
Levoglucosenone (**1**) (LGO) and presentation of two competitive approaches to the activated C=C bond of the *α*,*β*-unsaturated ketone unit.

In the recent decades (–)-levoglucosenone (**1**) (LGO, Figure 1) (systematic name: 1,6-anhydro-3,4-dideoxy-β-D-glycero-hex-3-enopyranos-2-ulose or (1S,5R)-6,8-dioxabicyclo [3.2.1]oct-2-en-4-one), obtained for the first time in 1979 by pyrolysis of cellulose in the presence of H_3_PO_4_ [1], attracts great attention as an exceptional chiral molecule and bioisoster useful for the design of diverse bioactive compounds [2,3,4,5,6] and for the construction of wireless data communication devices [7]. Due to the rapidly growing demand for large-scale applications, industrial methods for fabrication of **1**, including biotechnological processes, have also been developed and they are summarized in a recent review [8].

The presence of the α,β-unsaturated ketone unit allows **1** to be applied as a versatile reagent for cycloadditions and in recent years it was explored both in Diels–Alder reactions with cyclic and acylic dienes [9,10,11] as well as in 1,3-dipolar cycloadditions with nitrile imines [12,13], nitrile oxides [12], nitrones [12,14], and with diazomethane [15]. In a very recent publication, the first (8+2) higher order cycloaddition (HOC) of **1** with tropothione was also described as a diastereoselective process [16].

**Scheme 1:**
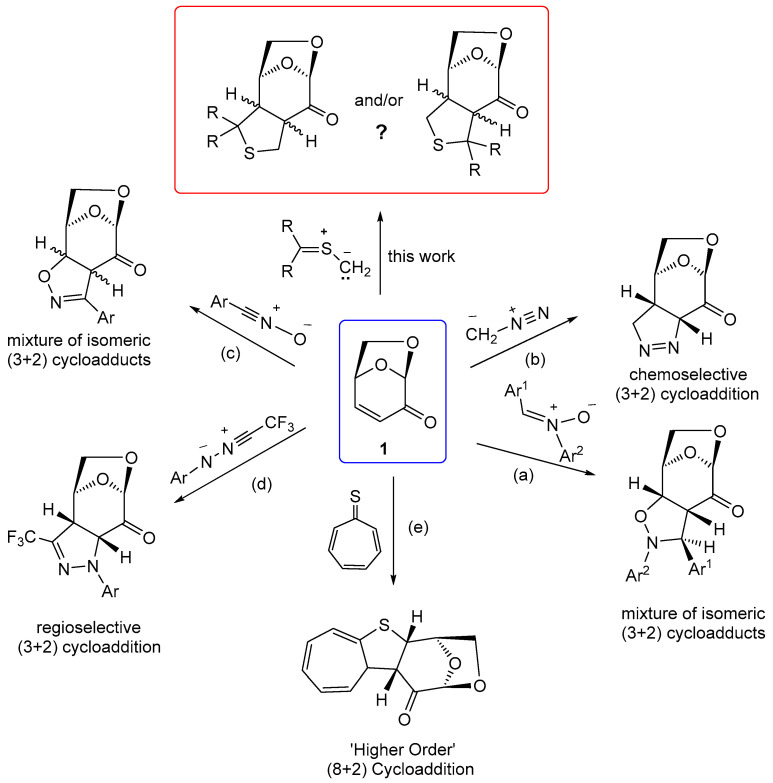
1,3-Dipolar cycloadditions of levoglucosenone (**1**) with: (**a**) nitrones [12]; (**b**) diazomethane [15]; (**c**) nitrile oxides [12]; (**d**) fluorinated nitrile imines [13]; and (**e**) (8+2) cycloaddition with tropothione [16].

The results of the (3+2) cycloadditions of **1** with various 1,3-dipoles, which are summarized in Scheme 1, demonstrate that the reported reactions occurred with different regio- and face-selectivity.

Notably, no (3+2) cycloaddition of **1** with any of the known *S*-centered 1,3-dipole (thiocarbonyl *S*-methanide, thiocarbonyl *S*-sulfide, thiocarbonyl *S*-oxide, or thiocarbonyl *S*-imide) has been reported to date.

**Scheme 2:**
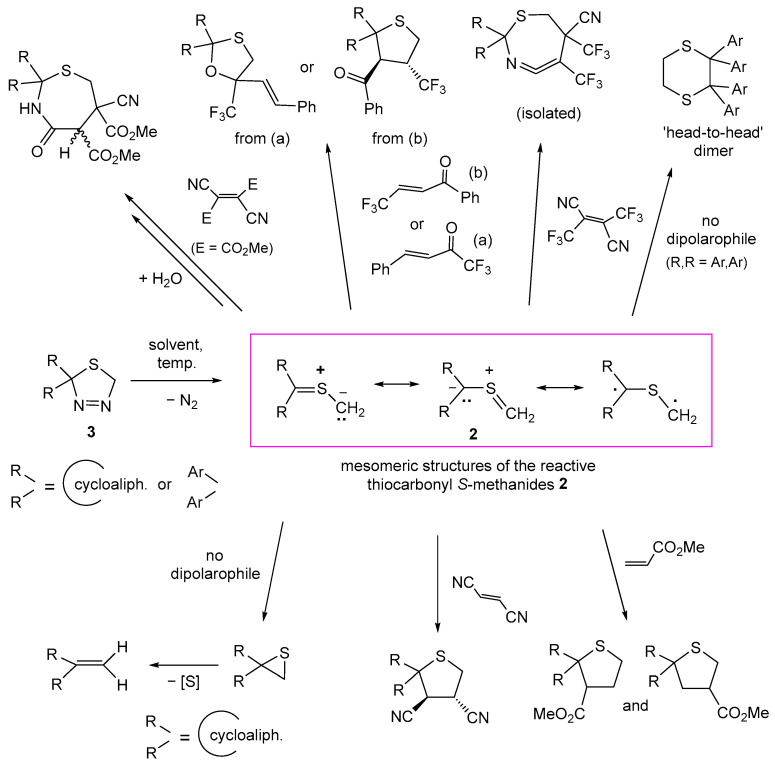
Selected 1,3-dipolar cycloadditions of aromatic, and cycloaliphatic thiocarbonyl *S*-methanides **2** with activated C=C dipolarophiles leading to five- and seven-membered cycloadducts, and their conversions observed in the absence of a dipolarophile yielding either thiirane (ring closure) or 1,4-dithiane derivatives.

Thiocarbonyl *S*-methanides **2** belong to the class of the electron-rich, so-called ‘*S*-centered 1,3-dipoles’, and their utility for a straightforward synthesis of various sulfur heterocycles was demonstrated in numerous publications [17,18]. In contrast to stable, push-pull-stabilized (and not reactive) thiocarbonyl ylides they can conveniently be generated as reactive intermediates via thermal decomposition of 1,3,4-thiadiazolines **3**, which are accessible via (3+2) cycloaddition of diazomethane and its derivatives with thiocarbonyl compounds such as thioketones or dithioesters (Scheme 2) [17,18,19].

In the presence of an activated C=C dipolarophile they are trapped according to the rules of (3+2) cycloadditions yielding the corresponding tetrahydrothiophene derivatives [17,18]. Interestingly, thiocarbonyl *S*-methanides **2** smoothly react with α,β-unsaturated ketones activated by the strongly electron-withdrawing CF_3_-group and the regioselectivity of the (3+2) cycloaddition depended on the localization of the CF_3_ group. The five-membered cycloadducts formed with complete regioselectivity as sole products in these reactions were identified as tetrahydrothiophene (from (b)) or 1,3-oxathiolane (from (a)) derivatives, respectively (Scheme 2) [20]. The intriguing regioselectivity observed in these reactions with enones bearing the strongly electron-withdrawing CF_3_ moiety was the subject of a recent, theoretical study which predicted the experimentally observed chemoselectivity [21].

Noteworthy, the *S*-CH_2_ terminus displays both basic and nucleophilic properties, which were demonstrated in some reactions of adamantanethione *S*-methanide (**2a**) [22]. On the other hand, in the absence of a reactive dipolarophile, thiocarbonyl *S*-methanides undergo an intramolecular cyclization to form thiiranes (as in the case of cycloaliphatic thiocarbonyl *S*-methanides) or dimerize in a characteristic, regioselective manner yielding sterically crowded tetraaryl-1,4-dithiane derivatives (as in the case of aromatic *S*-methanides, e.g., thiobenzophenone or thiofluorenone *S*-methanides) [23,24].

A mechanistically interesting, first case of a non-concerted, stepwise cycloaddition, was encountered in the reactions of sterically crowded thiocarbonyl *S*-methanides derived from cyclobutanethiones with strongly electron-deficient ethylenes such as dimethyl dicyanofumarate [25] or dimethyl 1,2-bis(trifluoromethyl)-1,2-di(methoxycarbonyl)ethylene [26,27]. Unexpectedly, in both cases seven-membered ketenimines were postulated as initial products of the stepwise (5+2) cycloaddition. Moreover, in the latter case, due to the stabilizing effect of the ‘magic’ CF_3_ group, the strained seven-membered ketenimine could be isolated and identified not only by means of spectroscopic methods, but also by the single-crystal X-ray diffraction analysis [27].

In spite of the great importance of α,β-unsaturated ketones as versatile synthons, widely applied in organic synthesis (Michael additions, electrophilic additions, cycloaddition reactions, etc.) [28], no applications for the synthesis of thiophene derivatives via (3+2) cycloadditions with thiocarbonyl *S*-methanides are known.

It is worth stressing that to date, in contrast to numerous C=C dipolarophiles presented in Scheme 2, no cycloadditions of thiocarbonyl *S*-methanides **2** with α,β-unsaturated ketones (except the CF_3_-group-activated representatives depicted in Scheme 2) have been reported.

The main goal of the present study was the examination of the reactivity of levoglucosenone (**1**), as a representative of non-enolizable, cyclic α,β-unsaturated ketones, towards thiocarbonyl *S*-methanides **2**, and the stereoselectivity (both regio- and face-selectivity) of the anticipated (3+2) cycloaddition was of special interest. For comparison reasons, the reactivity of some typical enones, like chalcone **4**, towards the in situ-generated thiocarbonyl *S*-methanides **2**, should also be tested.

## 2. Results and Discussion

Regardless of the fact that (3+2) cycloadditions of thiocarbonyl *S*-methanides **2** with diverse C=C dipolarophiles were widely studied and described in detail in numerous publications, e.g., [17,18], reactions with α,β-unsaturated ketones (enones) are limited to the CF_3_-activated derivatives presented in Scheme 2 [20,21]. For a better insight into the reactivity of thiocarbonyl *S*-methanides **2** towards non-activated enones, their reactions with chalcones should be examined within a preliminary study. The goal of this study was the examination of the reactivity of **2** towards typical enones and the influence of the steric hindrance in the 1,3-dipole on the course of the (3+2) cycloaddition was also of interest.

**Scheme 3:**
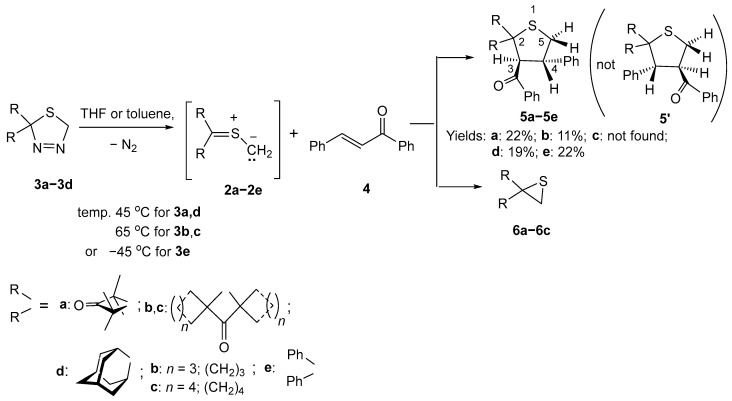
Regioselective (3+2) cycloadditions of in situ-generated thiocarbonyl *S*-methanides **2a**–**e** with chalcone **4** leading to cycloadducts **5**.

In the initial stage of the study, nearly equimolar amounts of chalcone **4** and the well-known *S*-methanide precursor **2a** (i.e., D^3^-2,5-dihydro-1,2,4-thiadiazoline **3a**) were applied to test the reaction performed under typical conditions, i.e., in THF solution at 45 °C (Scheme 3) [22,29].

After ca. 4h, the evolution of N_2_ was complete and after removal of the solvent, the crude product was analyzed by running the ^1^H NMR. Four equally intense singlets found at 0.92, 1.11, 1.22, and 1.34 ppm, along with two multiplets at 3.11–3.18 and 3.30–3.37 ppm, were attributed to the C*H*_2_ group, thereby suggesting the formation of a single cycloadduct. In addition, the presence of the doublet (^2^*J*_H,H_ = 6 Hz) found at 3.70 ppm, which could be attributed to *H*C(3) (but not to *H*C(4)), proved the presence of the regioisomer **5a** (and not the alternative **5’a**). Along with signals of cycloadduct **5a**, the ^1^H NMR also showed the distinct peaks of the known thiirane **6a,** which were found at 1.13 (*s*, 2Me), 1.22 (*s*, 2Me), and 2.59 (*s*, *H*_2_C) ppm, respectively. Based on comparison of the intensities of integration lines of the corresponding signals, the ratio of **5a** (minor) and **6a** (major) could be estimated to ca. 3:7. Thus, formation of thiirane **6a** as the major product demonstrated that the ring closure of the intermediate **2a** is faster than the (3+2) cycloaddition leading to **5a**. Chromatographic separation of the crude mixture of products led to isolation of the pure thiolane **5a** as a colorless, thick oil in 22% yield. Thiirane **6a** was isolated by PLC as a less polar fraction, contaminated with some amounts of the unconsumed chalcone.

Similar experiments with the sterically more crowded thiocarbonyl *S*-methanides **2b** and **2c** were performed at higher temperature (65 °C, toluene) (Scheme 3). These experiments showed that in the first case the amounts of the cycloadduct **5b** was substantially reduced (in comparison to **5a**) and the isolated yield of the pure compound was calculated to be 11% only. Finally, in the case of the sterically overcrowded **2c**, no (3+2) cycloaddition occurred, and the only product observed in this experiment was the corresponding thiirane **6c** [29].

In contrast, the attempted trapping of the in situ-generated adamantanethione *S*-methanide (**2d**) with chalcone **4** occurred with complete regioselectivity and led to the expected (3+2) cycloadduct **5d**. The ^1^H NMR analysis with a weighted portion of the concentration standard (1,1,2,2-tetrachloroethane) allowed determination of its yield to 38%. After chromatographic separation it could be isolated as a pure compound and subsequent crystallization from hexane/CH_2_Cl_2_ gave analytically pure, colorless crystals with m.p. 120–122 °C in 19% yield.

Finally, the trapping experiment with **4** using thiobenzophenone *S*-methanide (**2e**) was carried out at low temperature (−45 °C) following the known methodology elaborated some time ago by R. Huisgen et al. (Scheme 3) [30].

The yield of the (3+2) cycloadduct **5e** was also determined in the crude mixture after addition of a weighted portion of 1,1,2,2-tetrachloroethane and it was calculated to be 22%. However, in contrast to the earlier described experiments, neither crystallization nor chromatographic separation allowed isolation of **5e** in a pure form, without contamination with unconsumed chalcone **4**.

In a brief comment to this initial part of the study, one could conclude that, the in situ-generated thiocarbonyl *S*-methanides **2** display a moderate reactivity towards the C=C bond of chalcone **4** acting in these reactions as a model dipolarophile, representing the class of α,β-unsaturated ketones.

At ambient conditions, levoglucosenone (LGO) (**1**) exists as a thick oil, which is well soluble in organic solvents such as THF or toluene, and therefore all experiments with the in situ-generated 1,3-dipoles **2** could be performed under conditions applied for the reactions with the model chalcone **4** presented in Scheme 3. However, keeping in mind the moderate yields of the obtained/isolated cycloadducts **5**, the reactions with LGO **1** were carried out with precursors **3** used in a reasonable excess and the molar ratio of substrates **1** and **3** (LGO/precursor) was established at 1:3.

**Scheme 4:**
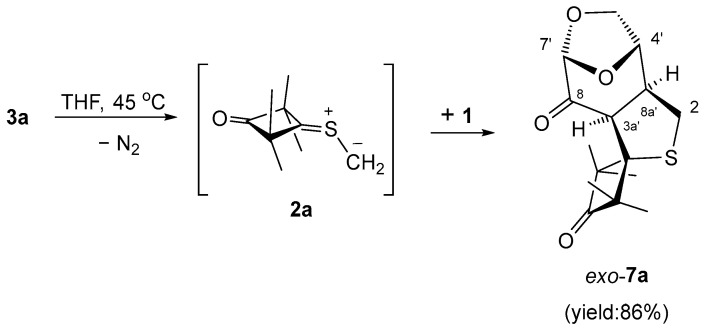
Complete stereoselectivity observed in the (3+2) cycloaddition of the in situ-generated thiocarbonyl S-methanide **2a** with levoglucosenone (**1**).

The first experiment of this series was performed in THF by heating **1** and **3a** at 45 °C. When the evolution of N_2_ was complete, the solvent was evaporated and the oily residue was analyzed by running the ^1^H NMR. The analysis of the registered signals revealed that **1** was completely consumed, and therefore, the characteristic singlet, existing in the ^1^H NMR spectrum (CDCl_3_ solution) of **1** at 5.80 ppm, could not be found. Instead, a new singlet appeared at 5.14 ppm and it could be attributed to the anticipated (3+2) cycloadduct **7a**. However, the best diagnostic signals [25] for its formation were two multiplets identified as *dd*, located at 3.15 and 2.80 ppm, attributed to the absorption of -*H*_2_C-S group of the tetrahydrothiophene ring. Importantly, there were no other signals observed which could suggest the formation of an isomeric product (Scheme 4).

**Figure 2:**
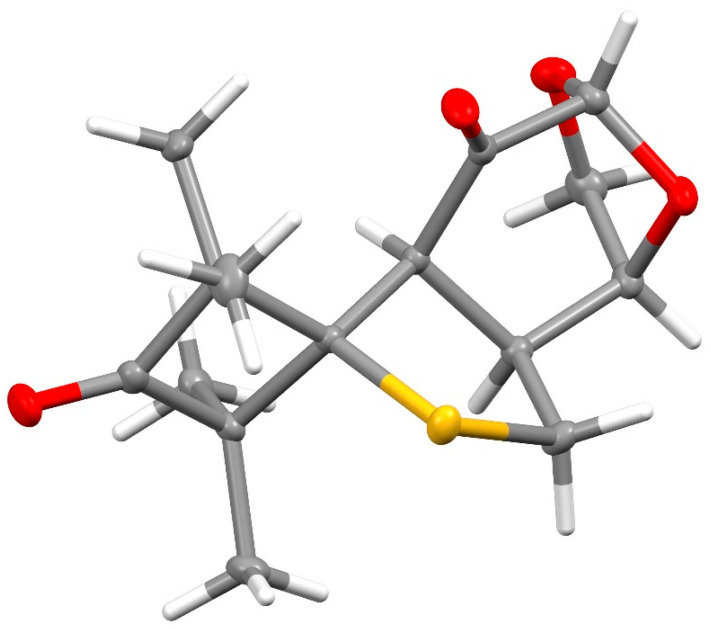
A view of the molecular structure of compound *exo*-**7a**. Displacement ellipsoids are drawn at the 50% probability level.

Isolation of the single (3+2) cycloadduct formed in this reaction was achieved by chromatography which delivered colorless crystals (86% yield) and the anticipated *exo*-**7a** structure was unambiguously confirmed by the X-ray diffraction analysis (Figure 2, Supplementary Material).

The conformation of the thiolane (tetrahydrothiophene, THT) ring has attracted the attention of researchers for a long time because it is incorporated in the structure of numerous natural compounds and pharmaceutically relevant organic compounds, e.g., biotin [31]. Therefore, its structural details were studied both in the gas phase as well as in the crystalline phase [32]. Whereas the twist conformation (*C*_2_) is preferred in the gas phase, the *C*_1_ symmetry (slightly distorted *C*_2_ symmetry) was reported for THT in the crystalline phase [33].

In the case of *exo*-**7a** and two more thiolanes *exo*-**7b**, and *exo*-**7d** (see Supplementary Material), which were characterized by X-ray measurement, the five-membered heterocyclic thiolane ring adopts the envelope conformation, with the carbon atom *C*(2) at the top, and the other four atoms lying in the common plane (Figure 1, Scheme 4). Apparently, this conformation results from the rigid structure of the levoglucosenone based, polycyclic system.

The correct attribution of signals of the H, and C atoms forming four stereogenic centers HC(7’), HC(4’), HC(3a’), and HC(8a’) was achieved based on the registered 2D spectra such as HMQC and COSY (see Supplementary Material, pp. 17, 18).

**Scheme 5:**
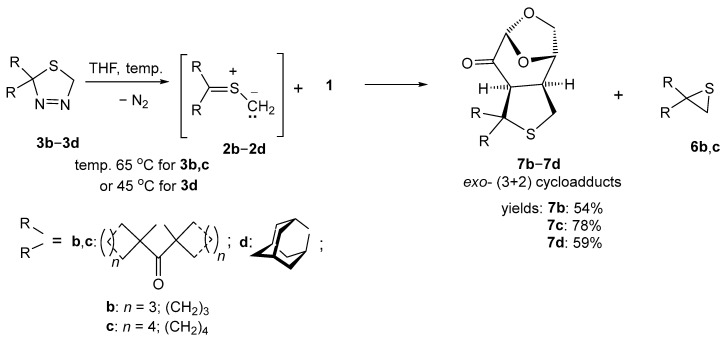
The stereoselective (3+2) cycloadditions of cycloaliphatic thiocarbonyl *S*-methanides **2b**-**2d** with levoglucosenone (**1**).

The successful experiment with **2a** prompted us to test other in situ-generated, more sterically crowded, thiocarbonyl *S*-methanides **2b**–**2d**, derived from cycloaliphatic thioketones as 1,3-dipoles, in analogous (3+2) cycloadditions with levoglucosenone (**1**). In order to check the influence of the steric hindrance on the outcome of the cycloaddition process, two bulky representatives bearing bis *spiro*-cyclopentyl and bis *spiro*-cyclohexyl rings, i.e., **3b** and **3c**, respectively, were tested in reactions performed in toluene at 65 °C. In an earlier study both precursors were shown to undergo decomposition slower than the tetramethyl substituted precursor **3a**, and therefore higher temperature was required to complete the N_2_ evolution from **3b** and **3c**, respectively, in a reasonable period of time [29]. To our surprise, the most sterically bulky 1,3-dipole **3c** afforded the expected (3+2) cycloadduct **7c** in high yield of 78%, and in the case of **3b**, the yield of isolated **7b** was slightly lower and calculated to be 54% (Scheme 5). A very comparable result was achieved starting with the precursor of adamantanethione *S*-methanide **2d**. In this case, the yield of isolated (3+2) cycloadduct was calculated to 59% (Scheme 5).

The ^1^H NMR spectra registered for the crude reaction mixtures proved the formation of single cycloadducts **7b**–**7d**, which after chromatographic isolation were crystallized from hexane/CH_2_Cl_2_ solutions yielding crystals suitable for the single-crystal X-ray diffraction analysis, and the anticipated *exo*-structure was unambiguously confirmed in all cases (see Supplementary Material).

Finally, two aromatic thiocarbonyl *S*-methanides **2e** and **2f**, derived from thiobenzophenone and thiofluorenone, respectively, were also used in the attempted (3+2) cycloadditions with levoglucosenone (**1**). It is well known, however, that in contrast to **2a**–**2d**, the precursors **2e** and **2f**, i.e., 1,3,4-thiadiazolines **3e** and **3f**, respectively, undergo decomposition at low temperature, and typically ca. −45 °C (acetone/dry ice bath) is recommended to arrange optimized reaction conditions [23,24].

**Scheme 6:**
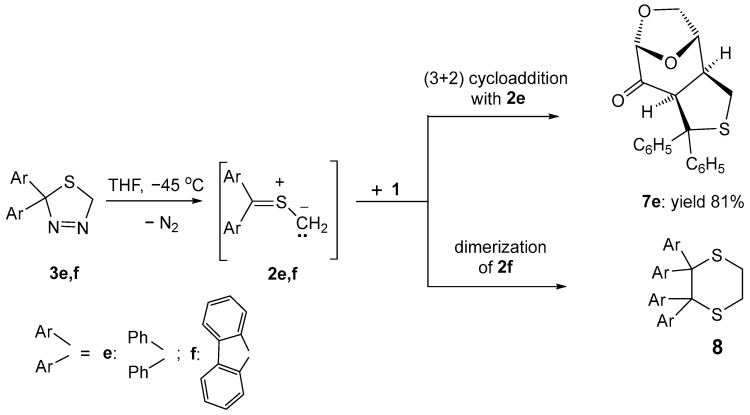
Attempted (3+2) cycloadditions of the in situ-generated, aromatic thiocarbonyl *S*-methanides **2e** and **2f** with levoglucosenone (**1**) led to different results.

The experiment with **3e** and **1** (molar ratio 3:1) afforded the expected cycloadduct **7e** as a single product which could be isolated chromatographically in 81% yield as a pure compound. The spectroscopic data proved its structure and by analogy to **7a**–**7d** the *exo*-orientation of the five-membered thiolane ring was also attributed to this polycyclic cycloadduct (Scheme 6).

Notably, chemical shifts of the *H* and *C* atoms located at stereogenic CH centers in cycloadducts *exo*-**7b**–**e** were nearly identical with those found for the cycloadduct *exo*-**7a**, and therefore the attribution of the respective signals to these atoms can be performed by analogy with the latter cycloadduct.

In contrast to **2e**, its analog **2f**, derived from thiofluorenone, did not react with **1** and instead underwent the so-called ‘head-to-head’ dimerization leading to the sterically crowded 1,4-dithiane **8** [23,30]. Apparently, under the applied reaction conditions, the (3+2) cycloaddition of **1** with **2f** is a slow process, and a fast dimerization process of the latter, via a postulated 1,6-diradical intermediate [23], is the dominating pathway.

**Scheme 7:**
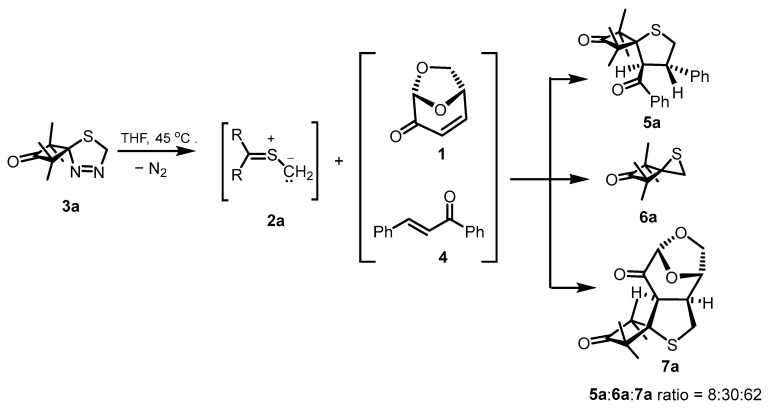
The competition experiment with the in situ-generated *S*-methanide **2a**, levoglucosenone (**1**), and chalcone **4** leading to a mixture of products **5a**, **6a** and **7a**.

In the final stage of the study a competition experiment comprising (3+2) cycloadditions of chalcone **4** and levoglucosenone (**1**) with the model thiocarbonyl *S*-methanide **2a** was carried out in THF solution at 45 °C. In this case, decomposition of the precursor **3a** (1 mol eq.) was performed in the presence of equimolar amounts of both dipolarophiles **1** and **4**. After 3h, evolution of N_2_ was complete, and after evaporation of the solvent the crude mixture of products was analyzed by ^1^H NMR. Characteristic signals found in the high-field region between 0.90 and 1.41 ppm could be attributed to the diagnostic Me groups of three components identified as the (3+2) cycloadducts **7a** (signals at 1.27, 1.31, 1.36, and 1.37 ppm) and **5a** (signals at 0.92, 1.11, 1.22, and 1.34 ppm), as well as the thiirane **6a** [25] (signals at 1.13, 1.22 ppm). Comparison of the intensities of well-separated CH_3_ signals clearly demonstrated that the molar ratio of compounds **5a**:**6a**:**7a** was ca. 8:30:62. Thus, under the applied reaction conditions, the LGO (**1**) was ca. eight times more reactive in the (3+2) cycloaddition with **2a** than the chalcone **4**. Notably, the ring closure of **2a** to form thiirane **6a** was also faster than its (3+2) cycloaddition with **4** (Scheme 7).

The mechanism of the (3+2) cycloadditions of the in situ-generated, reactive thiocarbonyl *S*-methanides **2** has intensively been discussed in the literature of the recent three decades, and one of the most intriguing reasons was the non-orthodox, stepwise mechanism in the case of reactions of electron-rich thiocarbonyl *S*-methanides with electron deficient ethylene dipolaropohiles [25,26,27].

**Scheme 8:**
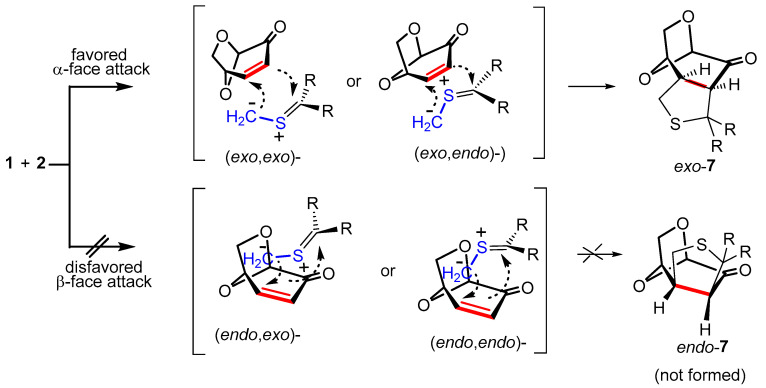
Transition states in the postulated mechanism of the (3+2) cyloadditions of levoglucosenone (**1**) with thiocarbonyl *S*-methanides **2.**

For the reported (3+2) cycloadditions of levoglucosenone (**1**) with thiocarbonyl *S*-methanides **2**, a concerted but rather non-synchronous mechanism can be postulated (Scheme 8).

The obtained results and the structure of isolated cycloadducts **7** demonstrated that in the transition state the *exo*-face attack of the 1,3-dipoles onto the C=C bond governs the course of the reaction. It is noteworthy that in all 1,3-dipoles (*S*-methanides) **2** used in the study, there are no prochiral centers at the terminal C atoms, and therefore, irrespective of the mode of the attack (*exo*,*exo*- or *exo*,*endo*-) (see Scheme 8), the finally formed (3+2) cycloadduct **7** exists as a single *exo*-oriented diastereoisomer.

## 3. Materials and Methods

**General information:** For general information see Supplementary Material.

*Reaction of 1,2-diphenylprop-3-en-2-one (**4**) with thiocarbonyl S-methanide* **2a:** A magnetically stirred solution of chalcone **4** (104 mg, 0.5 mmol) and 0.5 mmol (99 mg) of the precursor **3a** in dry THF (1 mL) was heated in an oil bath at 45 °C. Evolution of N_2_ was controlled using a bubbler placed at the top of a 5 mL round-bottomed flask. When the evolution of N_2_ was finished, the solvent was evaporated in vacuo, and the oily residue was examined by ^1^H NMR. After this control, the mixture of crude products was purified on preparative LC plates using a mixture of hexane and CH_2_Cl_2_ (1:1) as the eluent. Cycloadduct **5a** was isolated as viscous oil which solidified at r.t., and analytically pure samples were obtained by crystallization from hexane with a small admixture of CH_2_Cl_2_.


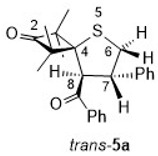


***trans*-8-Benzoyl-1,1,3,3-tetramethyl-7-phenyl-5-thiaspiro [3.4]octan-2-one (5a).** Yield: 42 mg (22%), colorless crystals, m.p. 117–119 °C (hexane/CH_2_Cl_2_).

**^1^H-NMR** (600 MHz, CDCl_3_): δ = 7.96 (d, ^3^*J*_H,H_ = 6 Hz, 2H, 2*H*C_ar_), 7.62–7.57 (m, 1H, *H*C_ar_), 7.52–7.46 (m, 2H, 2*H*C_ar_), 7.36–7.29 (m, 4H, 4*H*C_ar_), 7.28–7.23 (m, 1H, *H*C_ar_), 4.76 (d, ^3^*J*_H,H_ = 6 Hz, 1H, *H*C(8)), 3.72–3.67 (m,1H, *H*C(7)), 3.37–3.30, 3.19–3.12 (2m, 2H, *H*_2_C(6)), 1.34, 1.22, 1.11, 0.92 (4s, 4CH_3_) ppm.

**^13^C-NMR** (151 MHz, CDCl_3_): δ = 221.9, 200.2 (2*C*=O), 140.8, 136.6 (2*C*_ar_), 133.4, 127.4 (2H*C_ar_*), 129.0, 128.8, 128.4, 127.6 (for 8H*C_ar_*), 70.5, 66.2, 63.4 (3*C*), 60.4, (H_2_*C*(6), 54.1, 36.6 (2H*C*), 25.5, 23.2, 22.9, 21.4 (4*C*H_3_) ppm.

**EA**: C_24_H_26_O_2_S (378.53) calcd.: C 76.15, H 6.92, S 8.47; found: C 76.13, H 6.88, S 8.67.

*Reaction of levoglucosenone (***1***) with thiocarbonyl S-methanide* **2a**: A magnetically stirred solution of 1 (63 mg, 0.5 mmol) and 1.5 mmol (297 mg) of the precursor 3a in dry THF (2 mL) was heated in an oil bath at 45 °C. Evolution of N_2_ was controlled using a bubbler placed at the top of a 5 mL round-bottomed flask. After 3h evolution of N_2_ was finished; next, the solvent was evaporated in vacuo, and the oily residue was examined by running ^1^H NMR. After this control registration, the mixture of crude products was purified on the preparative LC plates using CH_2_Cl_2_ as an eluent. Cycloadduct 7a was isolated as a colorless solid. An analytically pure sample was obtained by crystallization from hexane with a small admixture of CH_2_Cl_2_.


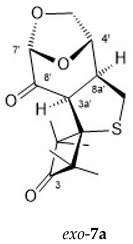


**(3a’*S*,4’*S*,7’*R*,8a’*R*)-2,2,4,4-Tetramethyl-3’*H*-spiro[cyclobutane-1,1’-[4,7]epoxythieno [3,4-*d*]oxepine]-3,8’(7’*H*)-dione** (cycloadduct *exo-***7a**). Yield: 128 mg (86%), colorless crystals, m.p. 170–171 °C (hexane/CH_2_Cl_2_).

**^1^H-NMR** (600 MHz, CDCl_3_): δ = 5.14 (s, 1H, *H*C(7’), 4.62 (m, 1H, *H*C(4’), 4.09–4.06 and 4.04–4.01 (2m, 2H), 3.69 (d, *J*_H,H_ = 12 Hz, *H*C(3a’)), 3.16–3.12, 2.97–2.92 (2m, 2H, S-C*H*_2_-), 2.82–2.76 (m, 1H, *H*C(8a’)), 1.39, 1.37, 1.32, 1.28 (4s, 4Me) ppm.

**^13^C-NMR** (151 MHz, CDCl_3_): δ = 219.5, 199.2, 102.1 (*C*7’), 74.8 (*C*4’), 68.5, 66.8, 65.1, 61.2, 52.9 (*C*3a’), 50.6 (*C*8a’), 30.9, 23.7 (Me), 21.8 (Me), 21.2 (Me), 21.1 (Me).

**EA**: C_15_H_20_O_4_S (296.38) calcd.: C 60.78, H 6.80, S 10.82; found: C 60.82, H 6.86, S 10.81.

α^20^_D_ = 77.86 [*c* = 0.3, CHCl_3_]

## 4. Conclusions

The study demonstrated new opportunities for exploration of LGO (**1**) as an exceptional dipolarophile, considered as a carbohydrate derivative, representing the class of the cyclic *endo*-enones. It extends the current knowledge on the hitherto reported reactivity of LGO [1,2,3,4,5,6,7,8,9,10,11,12,13,14,15,16,33]. The obtained results demonstrated for the first time, that LGO can efficiently be used as a trapping reagent (dipolarophile) towards the reactive, in situ-generated, electron-rich, thiocarbonyl *S*-methanides, derived from the non-enalizable, cycloaliphatic thioketones. The aromatic analogs can be explored to a limited extent, and only in the case of thiobenzophenone *S*-methanide (**2e**) a satisfactory result could be achieved. All of the studied (3+2) cycloadditions occurred in a highly stereoselective manner and the five-membered thiolane ring of the target product was formed with full regio- and *exo*-face selectivity.

The competition experiment performed with chalcone **4** and **1** proved that the latter, as a representative of α,β-unsaturated ketones, displays higher reactivity than its analog **4** towards electron-rich 1,3-dipoles such as thiocarbonyl *S*-methanides **2**. Very likely, ring strain in **1** contributes to an enhanced reactivity of the C=C bond.

In general, hitherto described methods aimed at synthesis of biologically active, sulfur-containing derivatives of levoglucosenone were focused on exploration of the thia-Michael addition of various thiols to the C=C group [34,35,36,37,38]. The now presented method of a new functionalization of the levoglucosenone skeleton with a tetrahydrothiophene ring may also be of interest in the future search for new, practically useful, bioactive glycomimetics [6,39] and fine reagents [8,40]. In addition, the described cycloadducts **7b**–**7d** possess in their structure spiro-centers which frequently determine biological activity of some polycyclic, organic compounds [41,42].

## Data Availability

All data are available in this publication. The original contributions presented in this study are included in the article/Appendix A.

## Supplementary Materials

The following supporting information can be downloaded at: https://www.mdpi.com/article/10.3390/molecules31132198/s1. Crystallographic data for this paper are provided free of charge by the joint Cambridge Crystallographic Data Centre: CCDC numbers for *exo*-**7b**, *exo*-**7d** and **7a** are 2368368, 2368369 and 2083333, respectively. The authors have cited additional references within the Supplementary Materials [22,29,30,43,44,45,46,47,48,49,50].

## Abbreviations

LGO	levoglucosenone
THF	tetrahydrofuran

## Appendix A. Entry for the Table of Contents

Levoglucosenone (LGO) **B** was, for the first time, reacted with the in situ-generated thiocarbonyl *S*-methanides **A** which belong to the class of the electron-rich, so-called ‘*S*-centered 1,3-dipoles’. The study showed that the expected (3+2) cycloadducts **C’** are formed in a highly stereoselective manner, but the yields of isolated products are moderate to satisfactory. Notably, the competition experiment performed with equimolar amounts of **B** and **C** demonstrated that **B** reacts with **A**, derived from 3-thioxo-2,2,4,4-tetramethylcyclobutanone, ca. eight times faster than 1,3-diphenylprop-2-enone (chalcone) **C**, which can be considered as a typical representative of α,β-unsaturated ketones.
