# Supplementary material for: Highly Stereoselective (3+2) Cycloadditions of Levoglucosenone (LGO) with the In Situ-Generated Thiocarbonyl S-Methanides (Thiocarbonyl Ylides) Derived from Aromatic and Cycloaliphatic Thioketones
Source: Molecules. 2026 Jun 23;31(13):2198. doi: 10.3390/molecules31132198 (PMC13363599; doi:10.3390/molecules31132198)
Supplement: Supplementary file 1 [file molecules-31-02198-s001.zip › molecules-4331656-supplementary.pdf]

Highly Stereoselective (3+2) Cycloadditions of Levoglucosenone (LGO) with the *in situ*-Generated Thiocarbonyl *S*-Methanides (Thiocarbonyl Ylides) Derived from Cycloaliphatic Thioketones#

Grzegorz Mlostoń,*[a], Małgorzata Celeda,[a] Marcin Palusiak,[b] Heinz Heimgartner,[c] Zbigniew J. Witczak[d]

[a] Prof. Dr. G. Mlostoń, M. Celeda
University of Łodź, Department of Organic & Applied Chemistry
Tamka 12, 91-403 Łodź (Poland)
E-mail: gregorz.mloston@chemia.uni.lodz.pl

[b] Prof. Dr. M. Palusiak
University of Łodź
Department of Physical Chemistry
Pomorska 163-165, 90-236 Łodź (Poland)

[c] Prof. Dr. Heinz Heimgartner
Department of Chemistry
University of Zurich
Winterthurerstrasse 190
CH-8057 Zürich (Switzerland)

[d] Prof. Dr. Zbigniew Witczak
Department of Pharmaceutical Sciences,
Nesbitt School of Pharmacy,
Wilkes University, 84 W. South Street, Wilkes-Barre,
PA 18766, USA

Table of Contents

1. Experimental p. S2
1.1. General Information
1.2 Starting Materials
1.3. Reactions of (*E*)-1,3-Diphenylprop-2-en-1-one (**4**) with Thiocarbonyl *S*-Methanides **2a**–**2e**
1.4. Attempted Reactions of Levoglucosenone (**1**) with Thiocarbonyl *S*-Methanides **2a**–**2f**
2. Collection of the ^{1}H and ^{13}C NMR Spectra p. S11
3. Crystal Structure Determination of Cycloadducts **7a**, **7b** and **7d** p. S28
4. References p. S30

1. Experimental

1.1. *General information*: Solvents and chemicals were purchased and used as received without further purification. Yields refer to isolated products. NMR spectra were recorded with a Bruker Avance III 600 MHz instrument (^{1}H NMR: 600 MHz; ^{13}C NMR: 151 MHz; ^{19}F NMR: 565 MHz). Chemical shifts are reported relative to solvent residual peaks (^{1}H NMR: δ = 7.26 ppm [$CHCl_3$]; ^{13}C NMR: δ = 77.0 ppm [$CDCl_3$]. The IR spectra were recorded with a Cary 630 FTIR (Agilent Technologies) spectrometer (as films). HRMS measurements were recorded on a Synapt G2 Si mass spectrometer (Waters) instrument. Specific rotations were determined on a Perkin-Elmer polarimeter at the sodium D line (589 nm). Optical rotations were determined in $CHCl_3$ solutions (at 20 ℃) with an Anton Paar MCP 500 polarimeter. Melting points were determined in capillaries with a Melt Temp II apparatus and they were not corrected. Products were purified by PLC dichloromethane or a mixture with hexane as eluents. Elemental analyses were performed at the Faculty of Chemistry Laboratory (University of Łódź) by Ms. Agnieszka Cieślińska and Hanna Jatczak using the C,H,N analyzer Vario EL III (Elemental Analysensysteme GmbH).

Numbering of atoms applied for the description of the NMR spectra of cycloadducts *exo*-**7b−e** results from systematic names of these compounds (see also main manuscript for *exo*-**7a**).

Commercial dichloromethane was dried by heating over $CaCl_2$ and freshly distilled prior to usage. Glass plates for preparative thin layer chromatography (PTLC) were coated with silica gel (Merck, Silica Gel 60 GF254, 5-40 μm mesh) in the laboratory by Ms. M. Celeda (University of Łódź) and activated prior to the usage in the laboratory oven at 100 °C. Exact reaction conditions are given in the following general procedures.

1.2. *Starting materials*: Levoglucosenone (**1**) was prepared by pyrolysis of cellulose according to the published procedure [S1]. Stable precursors of thiocarbonyl *S*-methanides **2a**–**d**, i.e. 1,3,4-thiadiazoline derivatives **3a**–**d**, were prepared following the known procedures: **2a** [S2], **2b** [S3], **2c** [S3], **2d** [S4]. The unstable 2,2-diphenyl-1,3,4-tiadiazoline (**2e**) was prepared from thiobenzophenone and diazomethane and

immediately used for the *in situ*-generation of **2e** [S5]. Chalcone was a commercial reagent purchased at Merck.

1.3. Reactions of 1,3-diphenylprop-2-en-1-one (4) with thiocarbonyl S-methanides 2b–2e – General Procedures:

a) *Starting with precursors* **3b**,**c**: A magnetically stirred solution of chalcone **4** (104 mg, 0.5 mmol) and 0.50 mmol of the corresponding precursor **3b** (125 mg) or **3c** (139 mg) in dry toluene (1 mL) was heated in an oil bath at 65 °C. Evolution of N_2 was controlled using a bubbler placed at the 5 mL round bottomed flask. When the evolution of N_2 was finished, the solvent was evaporated in vacuo, and the oily residue was examined by running ^{1}H NMR. After this control, the mixtures of crude products were purified on the preparative LC plates using a mixture of hexane and CH_2Cl_2 (7:3) as the eluent. The expected cycloadduct was isolated only in the case of **5b**. According to the ^{1}H NMR spectrum of the mixture of the crude products obtained from **3c**, only thiirane **6c** was formed and subsequently isolated by chromatography.

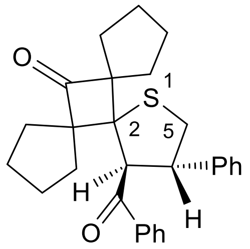

5b

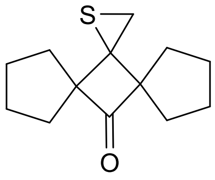

6b

***trans*-10-Benzoyl-9-phenyl-7-thiatrispiro[4.0.4^6.0.4^{11}.1^5]hexadecan-16-one (5b)**. Yield: 24 mg (11%), colorless oil.

^{1}H-NMR (600 MHz, $CDCl_3$): δ = 7.81-7.77 (m, 2H, 2HC$_{ar}$), 7.56-7.51 (m, 1H, HC$_{ar}$), 7.43-7.38 (m, 2H, 2HC$_{ar}$), 7.30-7.24 (m, 4H, 4HC$_{ar}$), 7.23-7.20 (m, 1H, HC$_{ar}$), 4.60 (d, $^3J_{H,H}$ = 6 Hz, 1H, HC(3)), 3.75-3.70 (m, 1H, HC(4)), 3.39-3.33, 3.24-3.18 (2m, 2H), 2.36-2.30 (m, 1H), 1.97-1.88 (m, 3H), 1.79-1.70 (m, 3H), 1.65-1.34 (m, 8H), 1.32-1.23 (m, 1H) ppm.

^{13}C-NMR (151 MHz, $CDCl_3$): δ = 219.8, 200.2 (2C=O), 140.7, 137.2 (2*C$_{ar}$*), 133.2 127.3 (2H*C$_{ar}$*), 128.8, 128.7, 128.0, 127.4 (for 8H*C$_{ar}$*), 76.4, 72.3, 68.9, 60.3, 50.0, 37.5, 37.1, 35.9, 34.4, 31.8, 27.1, 26.7, 26.3, 24.8 ppm.

HRMS (ESI) $C_{28}H_{30}O_2SNa$ requires 453.1864, found 453.1865 $[M+Na]^+$, 454.1898 [(M+1)+Na].

Monoisotopic Mass, Even Electron Ions
80 formula(e) evaluated with 2 results within limits (all results (up to 1000) for each mass)
Elements Used:
C: 0-40 H: 0-40 O: 0-5 S: 0-1 Na: 0-1

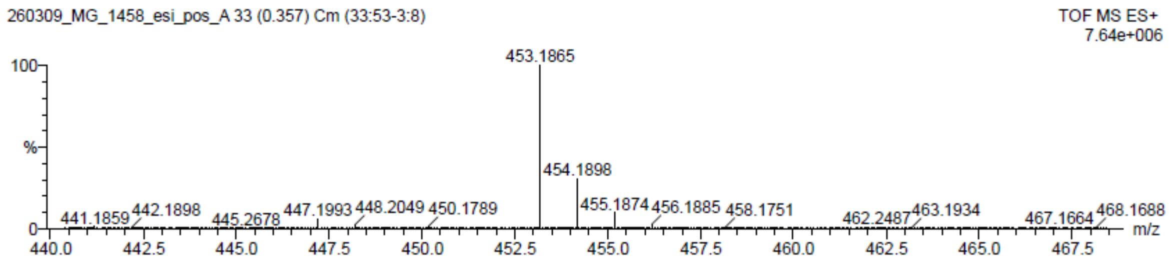

Minimum:				-1.5				
Maximum:		5.0	5.0	150.0				
Mass	Calc. Mass	mDa	PPM	DBE	i-FIT	Norm	Conf(%)	Formula
453.1865	453.1864	0.1	0.2	13.5	2236.7	0.000	100.00	C28 H30 O2 S Na
	453.1855	1.0	2.2	21.5	2247.6	10.827	0.00	C33 H25 O2

Fig. S1. The HRMS spectrum registered for **5b**.

1-Thiatrispiro[2.0.4^4.1.4^{10}.0^3]tetradecan-9-one (**6b**) [S3]: isolated after chromatography as a mixture with unconsumed chalcone **4**. Separation of both compounds was unsuccessful.

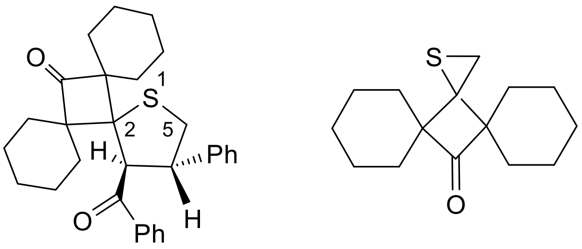

5c
(not formed)

6c

***trans*-4-Benzoyl-3-phenyl-1-thiatrispiro[4.0.5^6.1.5^{13}.0^5]octadecan-12-one (5c)**. This cycloadduct was not observed in the ^{1}H NMR registered for the crude products mixture.

1-Thiatrispiro[2.0.5^{4}.1.5^{11}.0^{3}]hexadecan-10-one (**6c**) [S3]: observed in a mixture with unconsumed chalcone **4**; identified by the presence of a characteristic singlet at 2.52 ppm.

b) *Starting with precursor* **3d**: A magnetically stirred solution of chalcone **4** (104 mg, 0.5 mmol) and 0.50 mmol of **3d** (104 mg) in dry THF (1 mL) was heated in an oil bath at 45 °C. Evolution of N_2 was controlled using a bubbler placed at the 5 mL round bottomed flask. When the evolution of N_2 was finished, the solvent was evaporated in vacuo, and the oily residue was examined by running ^{1}H NMR. After this control, the mixture of crude products was purified on the preparative LC plates using a mixture of hexane and CH_2Cl_2 (1:1) as the eluent. Cycloadduct **5d** was isolated as viscous, thick oil which solidified at r.t., and analytically pure samples were obtained by crystallization from hexane/CH_2Cl_2 (slow evaporation at r.t.).

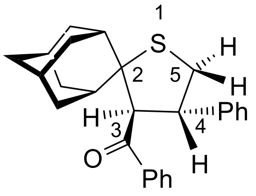

5d

***trans*-3'-Benzoyl-4'-phenylspiro[adamantane-2,2'-tetrahydrothiophene] (5d)**. Yield: 45 mg (19%), colorless crystals, m.p. 120–122 °C (hexane/CH_2Cl_2).

^{1}H-NMR (600 MHz, $CDCl_3$): δ = 7.72-7.67 (m, 2H, 2*H*C$_{ar}$), 7.47-7.42 (m, 1H, *H*C$_{ar}$), 7.35-7.29 (m, 2H, 2*H*C$_{ar}$), 7.27-7.21 (m, 4H, 4*H*C$_{ar}$), 7.19-7.13 (m, 1H, *H*C$_{ar}$), 4.30 (d, $^{3}J_{H,H}$ = 6 Hz, 1H, *H*C(3)), 3.87-3.81 (m,1H, *H*C(4)), 3.23-3.17 (m, 2H), 2.66-2.58, 2.44-2.38 (2m, 2H, H_2C(5)), 2.06-2.01 (m, 1H), 1.94-1.91 (m, 1H), 1.85-1.54 (m, 8H), 1.46-1.41 (m, 1H), 1.36-1.30 (1H) ppm.

^{13}C-NMR (151 MHz, $CDCl_3$): δ = 201.7 (C=O), 141.9, 138.2 (2*C$_{ar}$*), 132.7 127.0 (2H*C$_{ar}$*), 128.7, 128.6, 128.1, 127.2 (for 8H*C$_{ar}$*), 71.0, 62.2, 59.2, 39.9, 38.2, 36.7, 36.6, 35.9, 35.8, 35.4, 34.6, 26.6, 26.5 ppm.

EA: $C_{26}H_{28}OS$ (388.56) calcd.: C 80.37, H 7.26, S 8.25; found: C 80.29, H 7.14, S 8.20.

c) *Starting with precursor* **3e**: To magnetically stirred solution of thiobenzophenone (99 mg, 0.5 mmol) in 0.5 mL of dry THF placed in −75 °C cooling bath (acetone/dry ice), a cold solution of diazomethane in Et_2O was carefully added (dropwise) until the blue color of the thioketone completely vanished. Chalcone **4** (104 mg, 0.5 mmol) was added in portions. Magnetic stirring was continued and the cooling bath was allowed to warm. When the bath warmed to ca. −45 °C, a vigorous evolution of N_2 was observed. The bath was slowly further warmed to ca. 0 °C, the experiment was finished. The solvents were evaporated in vacuo, and the residual oil was examined by running ^1H NMR. The presence of the anticipated (3+2) cycloadduct **5e** was proved by the diagnostic signals at 5.15 (d, 1H), 4.42-4.34 (m, 1H), and 3.50-3.43 (m, 1H)/3.37-3.30 (m, 1H) ppm. After addition of a weighted portion of $Cl_2CHCHCl_2$ (46 mg, 0.27 mmol) a new registration of the ^1H NMR was carried out and the yield of **5e** could be determined in the mixture of crude products. The attempted separations of **5e** from unconsumed **4** and unidentified side products, were unsuccessful.

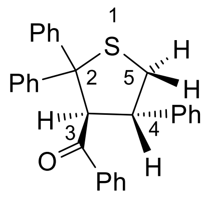

5e

***trans*-3-Benzoyl-2,2,4-triphenyltetrahydrothiophene** (**5e**). Yield: 22% (based on the ^1H NMR comparison with the weighed concentration standard.

1.4. Attempted reactions of Levoglucosenone (1) with Thiocarbonyl *S*-Methanides **2b−2e** – General Procedures:

a) *Starting with precursors* **3b**,**c**: A magnetically stirred solution of 1 (63 mg, 0.5 mmol), and 1.50 mmol of the corresponding precursor **3b** (375 mg) or **3c** (417 mg) in dry toluene (1 mL) was heated in an oil bath at 65 °C. Evolution of N_2 was controlled using a bubbler placed at the 5 mL round bottomed flask. When the evolution of N_2 was finished, the solvent

was evaporated in vacuo, and the oily residue was examined by running ^1H NMR. Next, the mixtures of crude products were purified on preparative LC glass plates coated with silica using a mixture of hexane and CH_2Cl_2 (7:3) as the eluent. The expected cycloadducts were isolated as viscous oils. Analytically pure samples were obtained by crystallization.

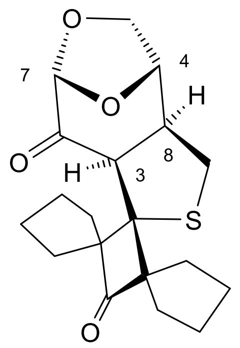

exo-**7b**

(3*S*,4*S*,7*R*,8*R*)-2,2,4,4-Bis(spirocyclopentane-1',2,1',4-tetrahydro-3''*H*-spiro[cyclobutane-1,1''-[4,7]epoxythieno[3,4-*d*]oxepine]-3,8''(7''*H*)-dione (cycloadduct (*exo*-**7b**)). Yield: 94 mg (54%), colorless crystals, m.p. 148–150 °C (hexane/CH_2Cl_2).

^{1}H-NMR (600 MHz, $CDCl_3$): δ = 5.12 (s, 1H), 4.62-4.58 (m, 1H), 4.09-4.05; 4.04-4.00 (2m, 2H), 3.57 (d, $J_{H,H}$ = 12 Hz, 1H), 3.28-3.22 (m, 1H), 3.03-2.97; 2.78-2.72 (2m, 2H), 2.20-2.08 (m, 2H), 1.92-1.60 (m, 13H), 1.58-1.50 (m, 1H) ppm.

^{13}C-NMR (151 MHz, $CDCl_3$): δ = 219.2, 198.9 (2C=O), 102.1, 76.3, 74.6, 69.6, 68.5, 64.9, 52.7, 50.0, 35.7, 33.2, 33.1, 31.9, 31.4, 26.9, 26.4, 26.3, 24.7 ppm.

EA: $C_{19}H_{24}O_4S$ (348.45) calcd.: C 65.49, H 6.94, S 9.20; found: C 65.50, H 6.51, S 9.20.

α_D^{20} = 120.38 [*c* = 0.3, $CHCl_3$]

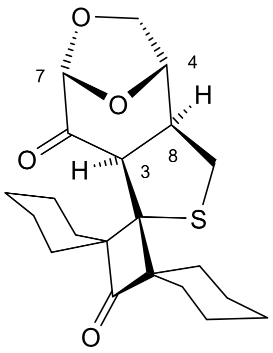

exo-**7c**

(3*S*,4*S*,7*R*,8*R*)-2,2,4,4-Bis(spirocyclohexane-1‘,2,1’,4-tetrahydro-3''*H*-spiro[cyclobutane-1,1''-[4,7]epoxythieno[3,4-*d*]oxepine]-3,8''(7''*H*)-dione (cycloadduct (*exo*-**7c**)). Yield: 147 mg (78%), colorless crystals, m.p. 230–232 °C (hexane/CH_2Cl_2).

^{1}H-NMR (600 MHz, $CDCl_3$): δ = 5.11 (s, 1H), 4.58 (d, $^3J_{H,H}$ = 2 Hz), 4.11-4.06, 4.04-3.97 (2m, 2H), 3.71 (d, $^3J_{H,H}$ = 12 Hz, 1H), 3.08-3.02; 2.90-2.83 (2m, 2H), 2.82-2.75; 2.73-2.67 (2m, 2H), 2.19-2.12 (m, 1H), 1.99-1.41 (m, 15H), 1.22-1.12 (m, 1H), 1.04-0.94, 0.92-0.85 (2m, 2H) ppm.

^{13}C-NMR (151 MHz, $CDCl_3$): δ = 218.2, 200.2 (2C=O), 102.3, 74.2, 68.6, 67.8, 67.5, 64.2, 52.0, 50.7, 32.7, 32.6, 31.8, 31.4, 29.9, 25.7, 25.4, 23.9, 23.7, 23.5, 23.2 ppm.

EA: $C_{21}H_{28}O_4S$ (376.51) calcd.: C 66.99, H 7.49, S 8.52; found: C 66.91, H 7.48, S 8.48.

α_D^{20} = 80.58.38 [*c* = 0.3, $CHCl_3$]

b) *Starting with precursor* **3d**: A magnetically stirred solution of levoglucosenone (**1**) (63 mg, 0.5 mmol) and 1.5 mmol of 3d (312 mg) in dry THF (1 mL) was heated in an oil bath at 45 ℃. Evolution of N_2 was controlled using a bubbler placed at the 5 mL round bottomed flask. When the evolution of N_2 was finished, the solvent was evaporated in vacuo, and the oily residue was examined by running ^{1}H NMR. After this control, the mixture of crude products was purified on preparative LC plates using a

mixture of hexane and CH_2Cl_2 (1:1) as the eluent. Cycloadduct **5d** was isolated as viscous, thick oil which solidified at r.t., and analytically pure samples were obtained by crystallization from hexane/CH_2Cl_2 (slow evaporation at r.t.).

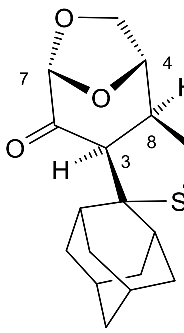

exo-**7d**

(3*S*,4*S*,7*R*,8*R*)-Tetrahydro-3'*H*-spiro[adamantane-2,1'-[4,7]epoxythieno[3,4-d]oxepin]-8'(7'*H*)-one (cycloadduct **7d**). Yield: 90 mg (59%), colorless crystals, m.p. 231–233 °C (hexane/CH_2Cl_2).

^{1}H-NMR (600 MHz, $CDCl_3$): δ = 5.04 (s, 1H), 4.62-4.59 (m, 1H), 4.05-4.01, 3.99-3.95 (2m, 2H), 3.66 (d. $^3J_{H,H}$ = 6 Hz, 1H), 3.26-3.22; 3.06-3.00 (2m, 2H), 3.17-3.12; 2.95-2.90 (2m, 2H), 2.47-2.42 (m, 1H), 1.93-1.87 (m, 1H), 1.83-1.77 (m, 4H), 1.74-1.61 (m, 7H) ppm.

^{13}C-NMR (151 MHz, $CDCl_3$): δ = 200.3 (C=O), 102.5, 74.9, 68.3, 67.6, 51.8, 49.5, 37.9, 37.3, 36.6, 35.5, 35.2, 33.9, 33.7, 30.2, 26.9, 26.4 ppm.

EA: $C_{17}H_{22}O_3S$ (306.42) calcd.: C 66.63, H 7.24, S 10.46; found: C 66.72, H 7.39, S 10.42.

α_D^{20} = −10.50 [*c* = 0.3, $CHCl_3$]

c) *Starting with precursors* 3e,f: To a magnetically stirred solution of 1.5 mmol the corresponding thioketone (thiobenzophenone (3e), 297 mg, or thiofluorenone (3f), 294 mg) in 0.5 mL of dry THF placed in a −75 °C cooling bath (acetone/dry ice), a cold solution of diazomethane in Et_2O

was carefully added (drop-wise) until the intense color of the thioketone completely vanished. Next, a portion of levoglucosenone (1) (63 mg, 0.5 mmol) was added to the stirred solution at −75 ℃ in small portions. Magnetic stirring was continued and the cooling bath was slowly allowed to warm. At ca. −45 °C, a vigorous evolution of N_2 was observed and the bath was slowly warmed to ca. 0 °C. At this temp. no evolution of N_2 was observed. Both experiments were finished at 0 °C, and subsequently the solvent was evaporated in vacuo. The oily residues were initially examined by running ^{1}H NMR. The presence of the anticipated (3+2) cycloadduct 7e was confirmed by the presence of diagnostic signals found at 5.10 (s, 1H), 4.06-4.02 (d, 1H), and 3.98-3.94 (dd, 1H) ppm. It could be isolated as a coloreless viscous oil by PLC on glass plates coated with silica. An analytically pure sample was obtained by crystallization.

The ^{1}H NMR of the mixture of the crude products obtained from 1 and 3f did not reveal the presence of the expected (3+2) cycloadduct 7f, but instead, a distinct singlet found at 5.28 ppm, suggested the presence of the unconverted 1. In addition, two multiplets found at 4.10-4.16 and 3.11-3.17 ppm could be attributed to the known thiofluorenone dimer 8. After preparative layer chromatography on the glass plates coated with silica, a colorless solid was isolated as the sole compound and identified as the known dimer of thiofluorenone *S*-methanide, i.e. 1,4-dithiane 8.

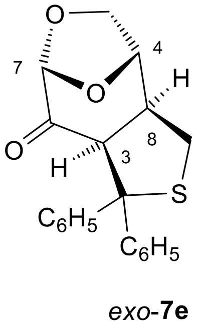

exo-**7e**

(3*S*,4*S*,7*R*,8*R*)-1,1-Diphenylhexahydro-4,7-epoxythieno[3,4-d]oxepin-8(7*H*)-one (*exo*-**7e**). Yield: 84 mg (50%), colorless crystals, m.p. 169–171 °C ($MeOH/CH_2Cl_2$).

^{1}H-NMR (600 MHz, $CDCl_3$): δ = 7.70 (d, $^3J_{H,H}$ = 12 Hz, 2H), 7.34-7.10 (m, 8H), 5.10 (s, 1H), 4.57-4.54 (m, 1H), 4.44 (d, $^3J_{H,H}$ = 6Hz, 1H), 4.06-4.02; 3.98-3.94 (2m, 2H), 3.28-3.23; 3.04-2.99 (2m, 2H), 2.87-2.81 (m, 1H) ppm.

^{13}C-NMR (151 MHz, $CDCl_3$): δ = 197.7 (C=O), 145.4, 143.0 (2C_{ar}), 128.6 127.9, 126.8, 126.2 (4HC_{ar}), 126.6, 126.2 (2 HC_{ar}), 102.0, 75.2, 68.9, 68.4, 56.1, 49.2, 30.8 ppm.

EA: $C_{20}H_{18}O_3S$ (338.42) calcd.: C 70.98, H 5.36, S 9.47; found: C 70.81, H 5.54, S 9.37.

α_D^{20} = 28.56 [*c* = 0.3, $CHCl_3$]

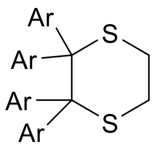

8

Dispiro[1,4-dithiane-2,9';3,9''-bis(fluorene)] (8). Yield: 139 mg (44%), colorless crystals, m.p. 276-278°C (lit. [S5], m.p . 250 °C (decomp.)).

^{1}H-NMR (600 MHz, $CDCl_3$): δ = 9.28-9.23 (m, 2H), 7.66-7.61 (m, 2H), 7.52-7.46 (m, 4H), 7.36 (d, $^3J_{H,H}$ = 6 Hz, 2H), 7.06-7.00, 6.70-6.64 (2m, 4H), 6.21 (d, $^3J_{H,H}$ = 12 Hz, 2H), 4.17-4.08, 3.18-3.10 (2m, 4H) ppm.

^{13}C-NMR (151 MHz, $CDCl_3$): δ = 149.6, 144.0, 140.2, 139.6 (4C_{ar}), 128.3, 128.2, 127.7, 126.5, 126.3, 125,2, 120.2, 119.1 (8HC_{ar}), 55.3 (2C_q), 27.3 (2H_2C) ppm.

EA: $C_{28}H_{20}S_2$ (420.60) calcd.: C 79.96, H 4.79, S 15.25; found: C 79.98 H 4.65, S 5.17.

2. **Collection of the ^{1}H, and ^{13}C NMR Spectra**

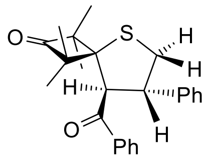

5a

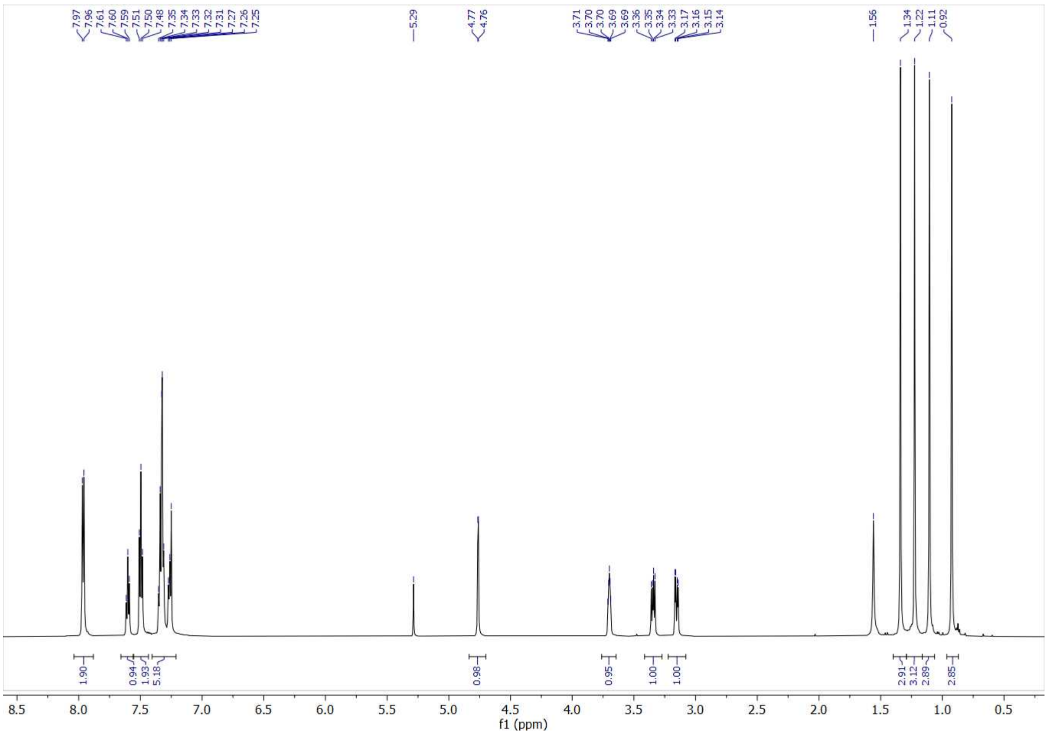

Fig. S2. ^{1}H NMR of the cycloadduct **5a**.

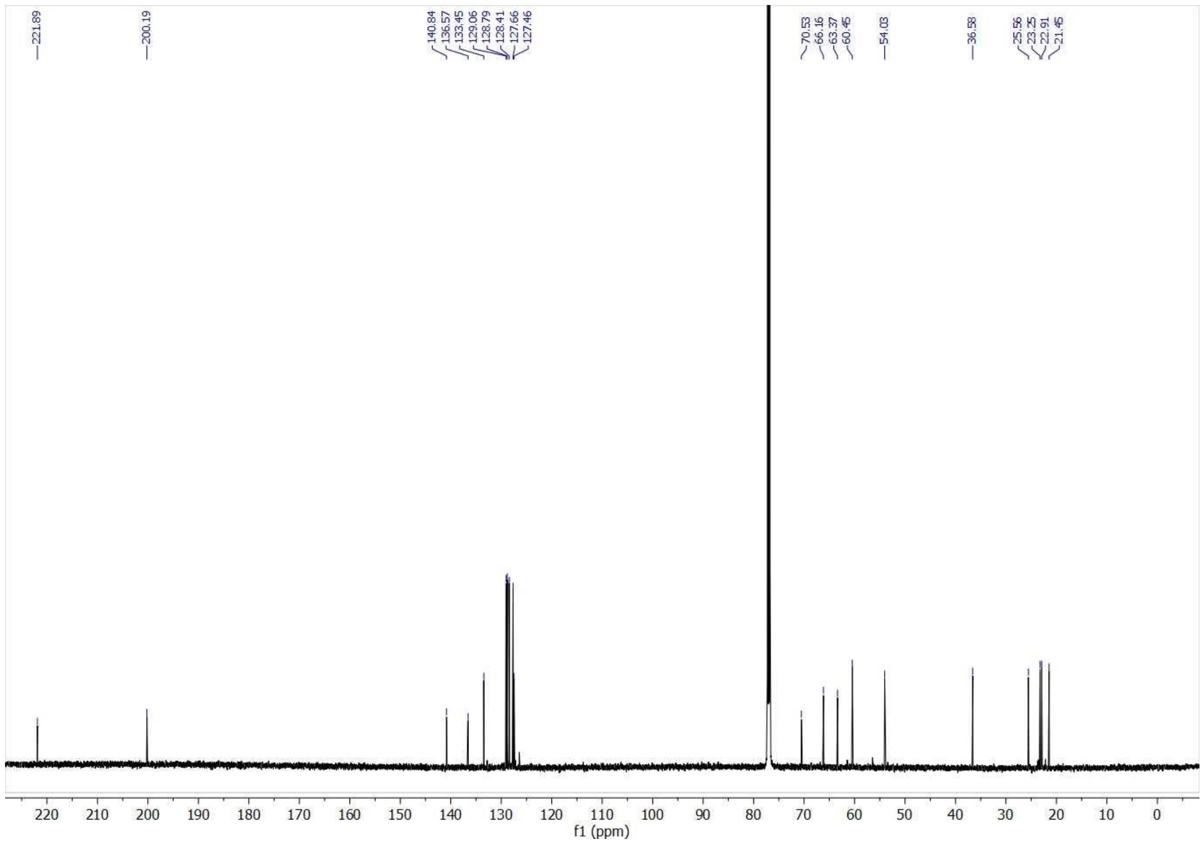

Fig. S3. ^{13}C NMR of the cycloadduct **5a**.

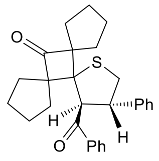

5b

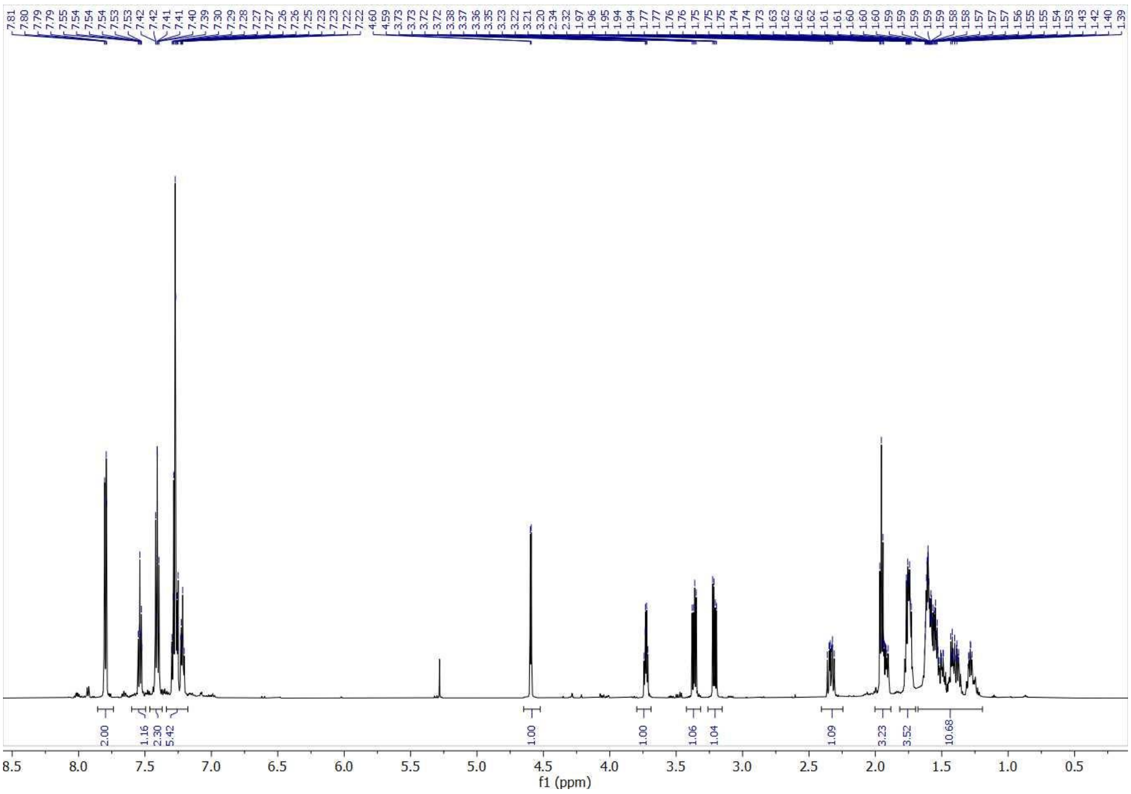

Fig. S4. ^{1}H NMR of the cycloadduct **5b**.

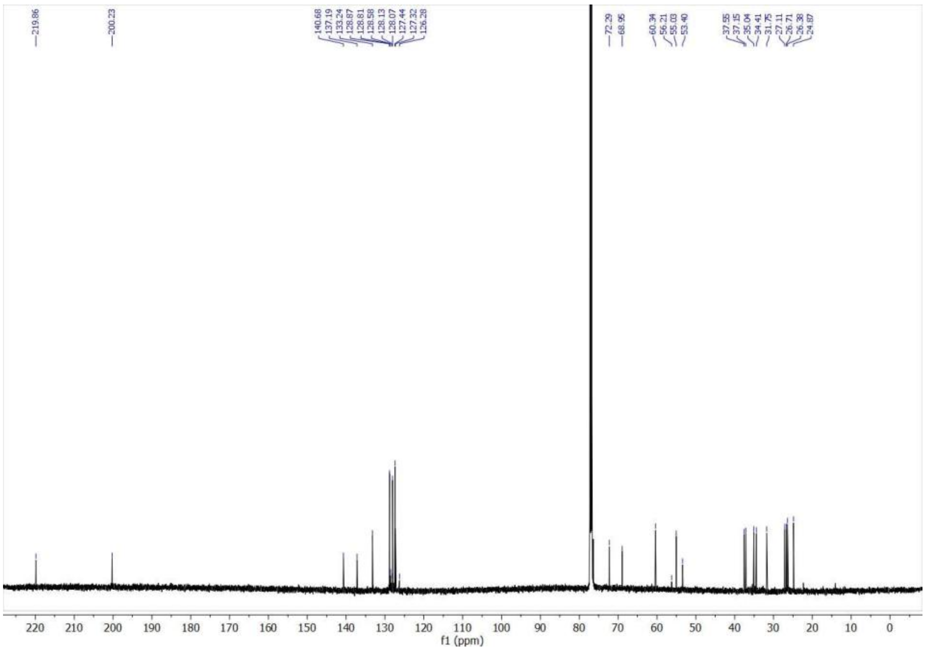

Fig. S5. ^{13}C NMR of the cycloadduct **5b**.

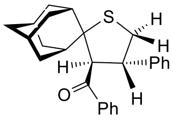

5d

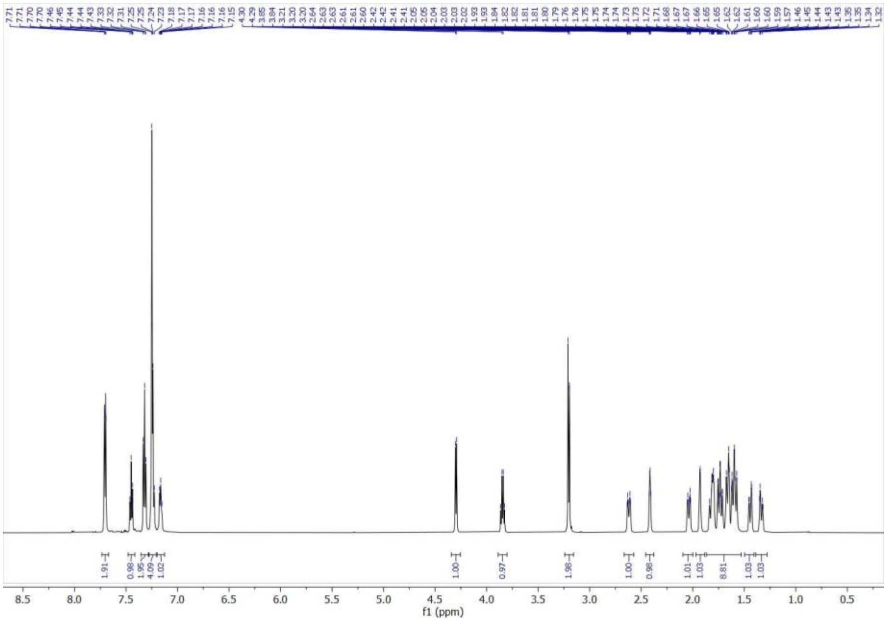

Fig. S6. ^{1}H NMR of the cycloadduct **5d**.

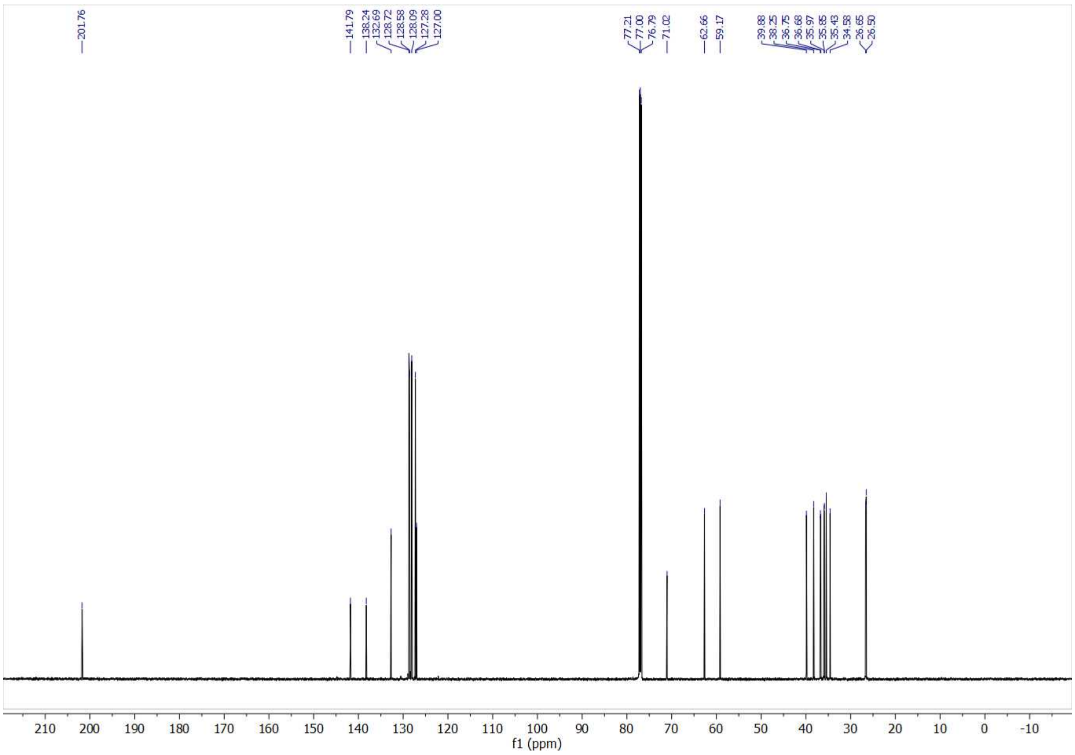

Fig. S7. ^{13}C NMR of the cycloadduct **5d**.

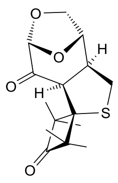

exo-**7a**

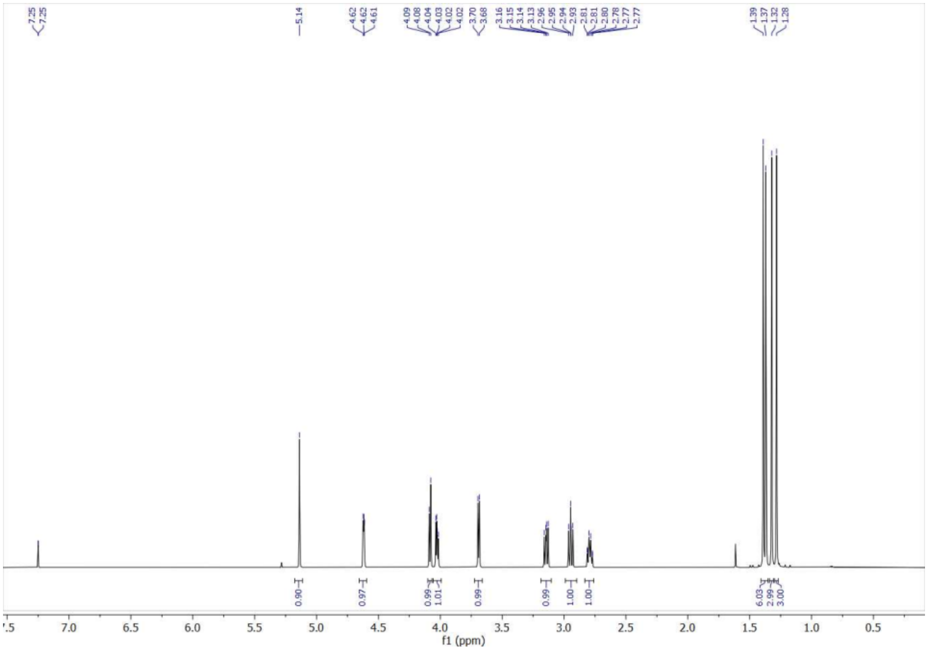

Fig. S8. ^{1}H NMR of the cycloadduct **7a**.

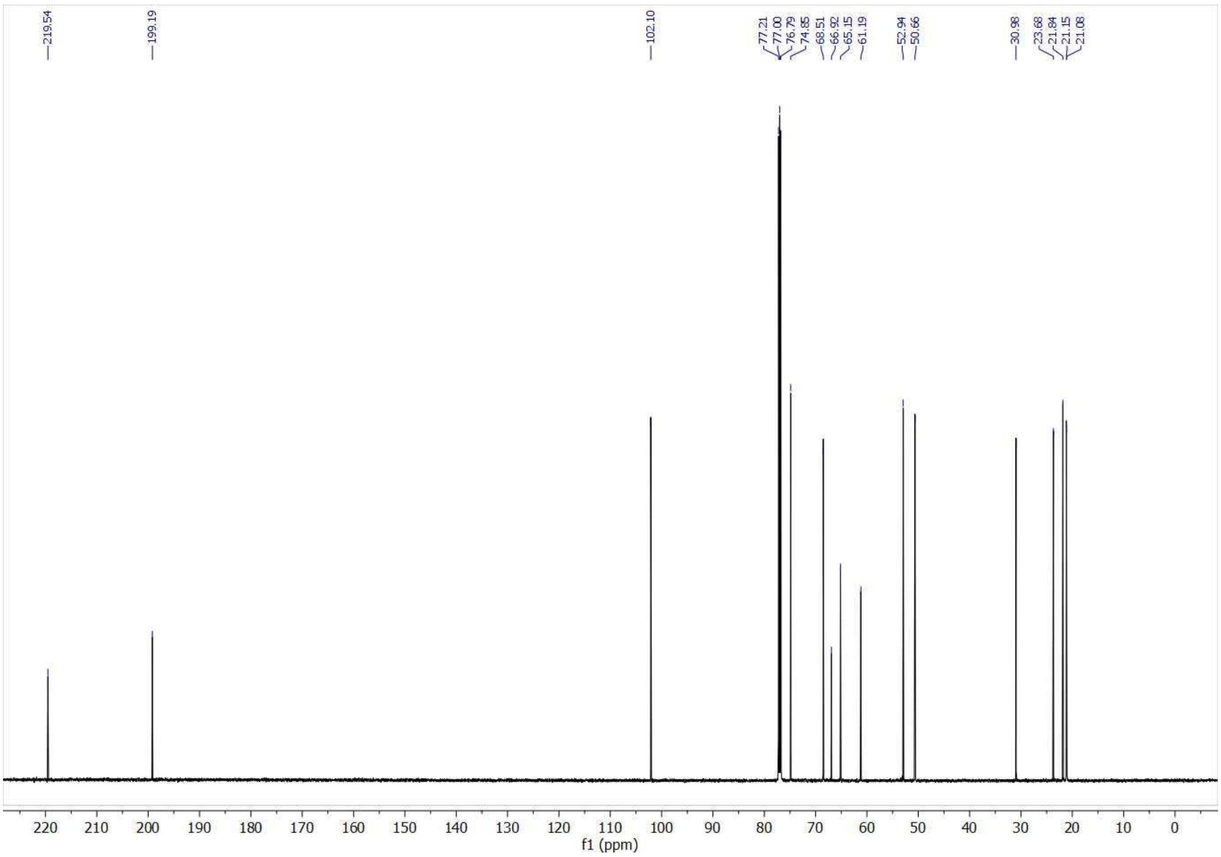

Fig. S9. ^{13}C NMR of the cycloadduct **7a**.

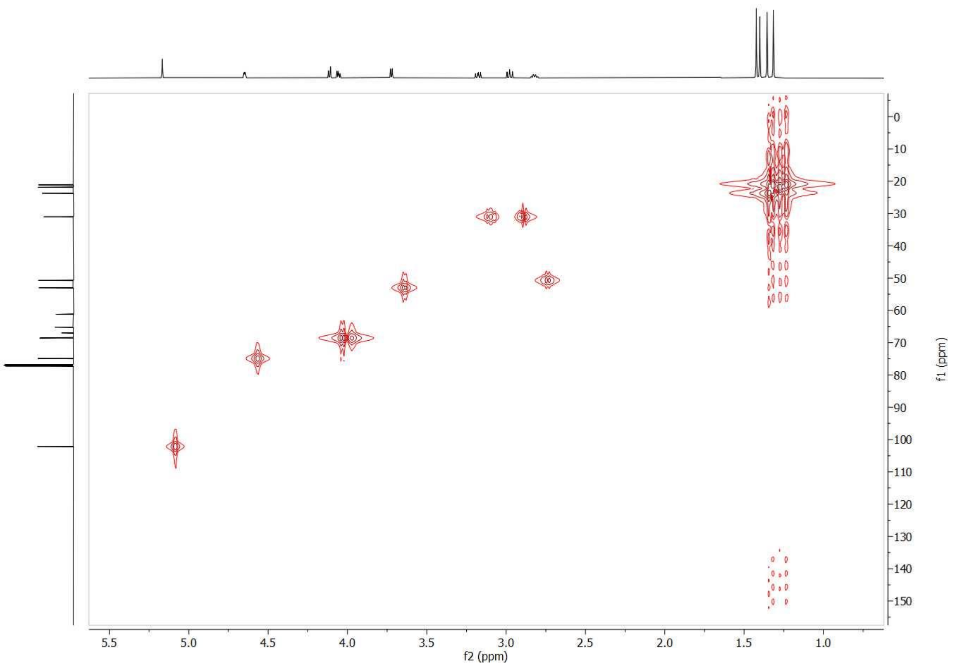

Fig. S10. The HMQC spectrum of the cycloadduct **7a**.

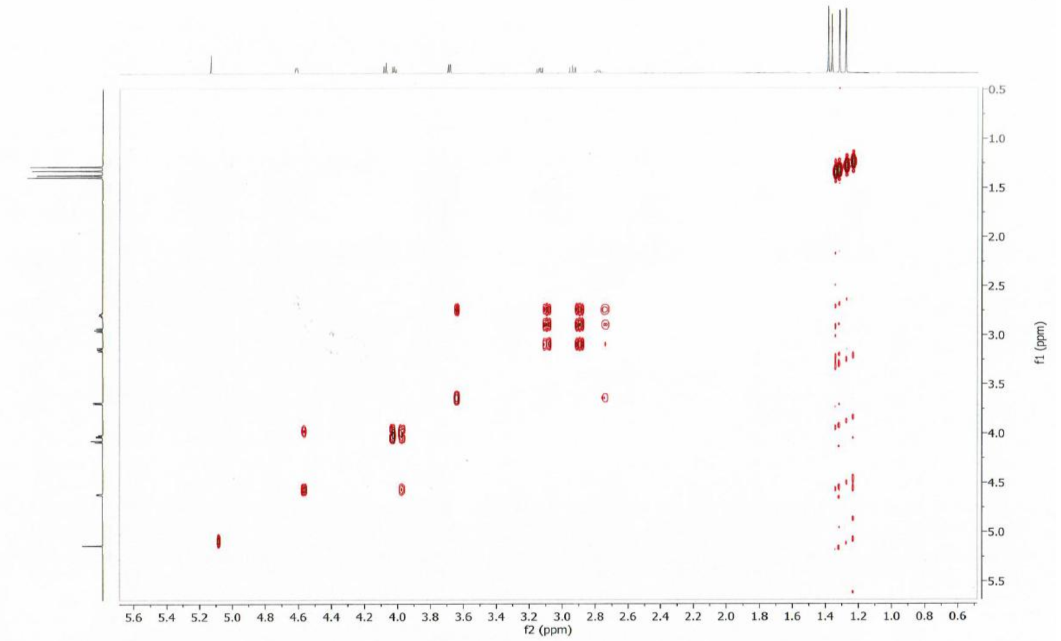

Fig. S11. The COSY spectrum of the cycloadduct **7a**.

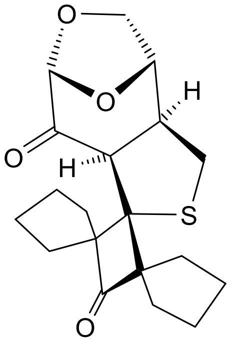

exo-**7b**

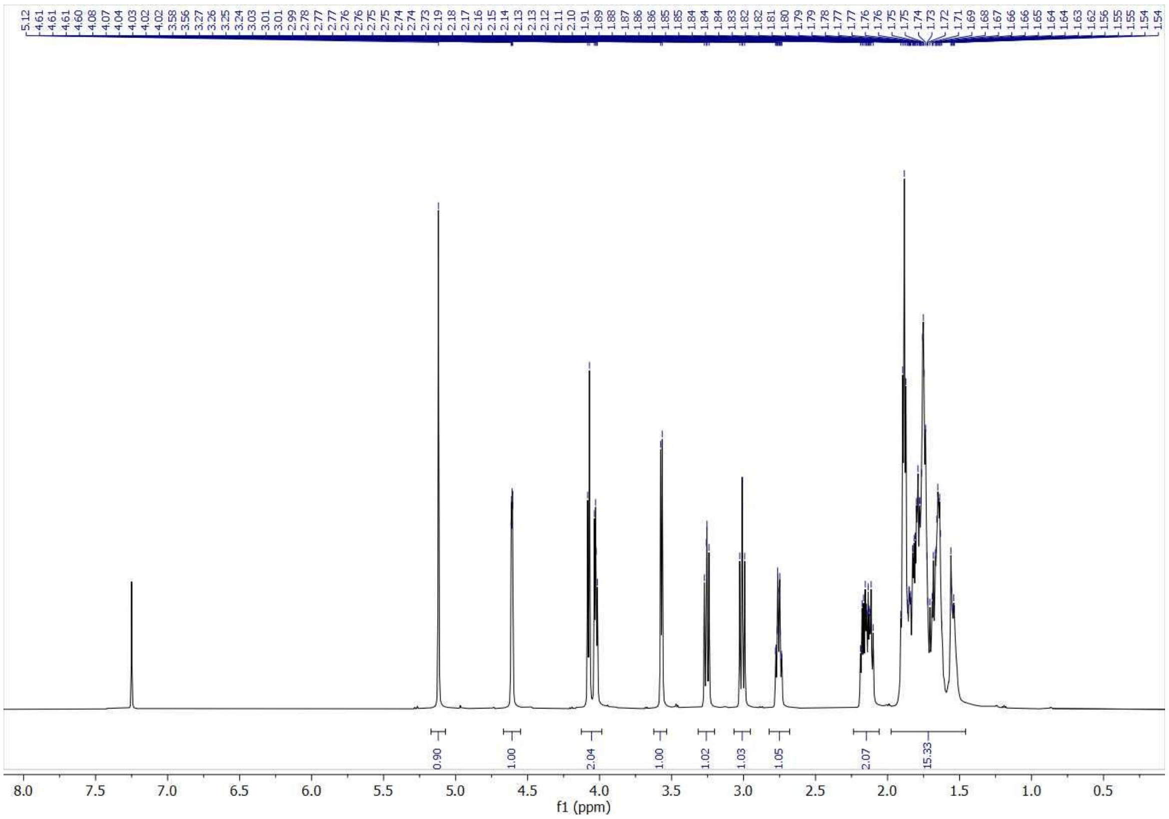

Fig. S12. ^{1}H NMR of the cycloadduct **7b**.

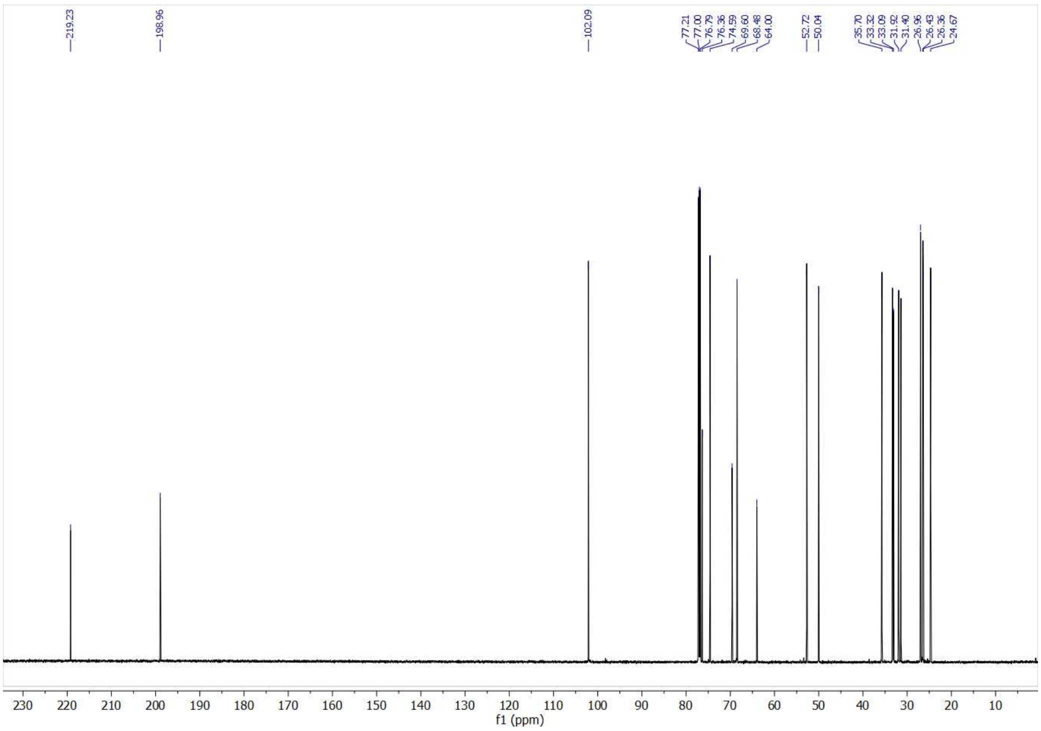

Fig. S13. ^{13}C NMR of the cycloadduct **7b**.

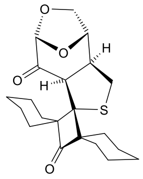

exo-**7c**

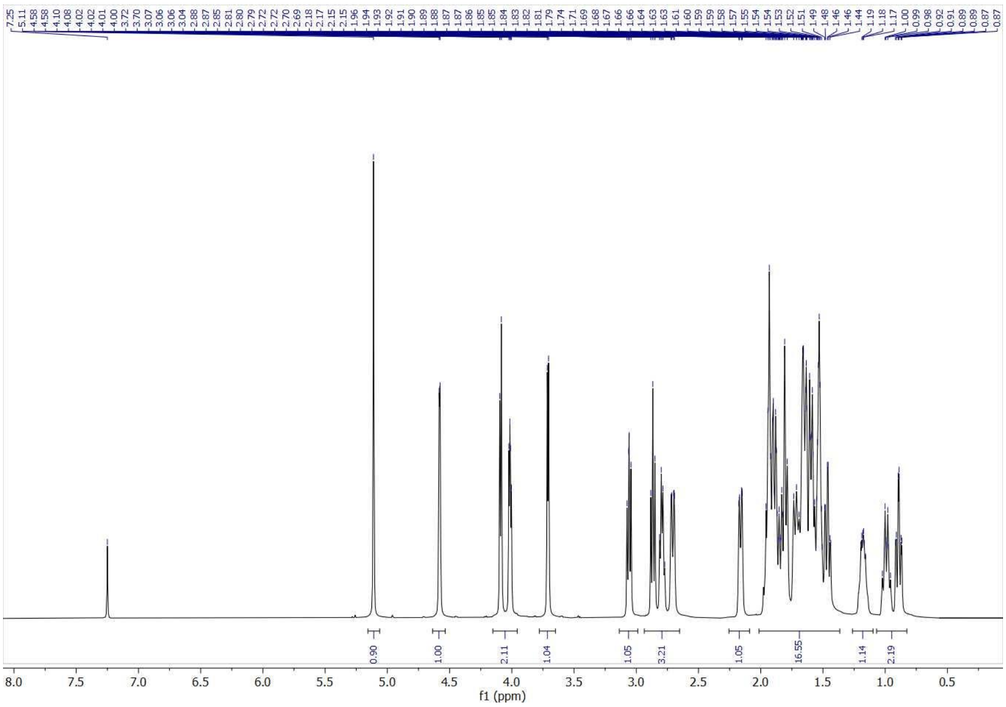

Fig. S14. ^{1}H NMR of the cycloadduct **7c**.

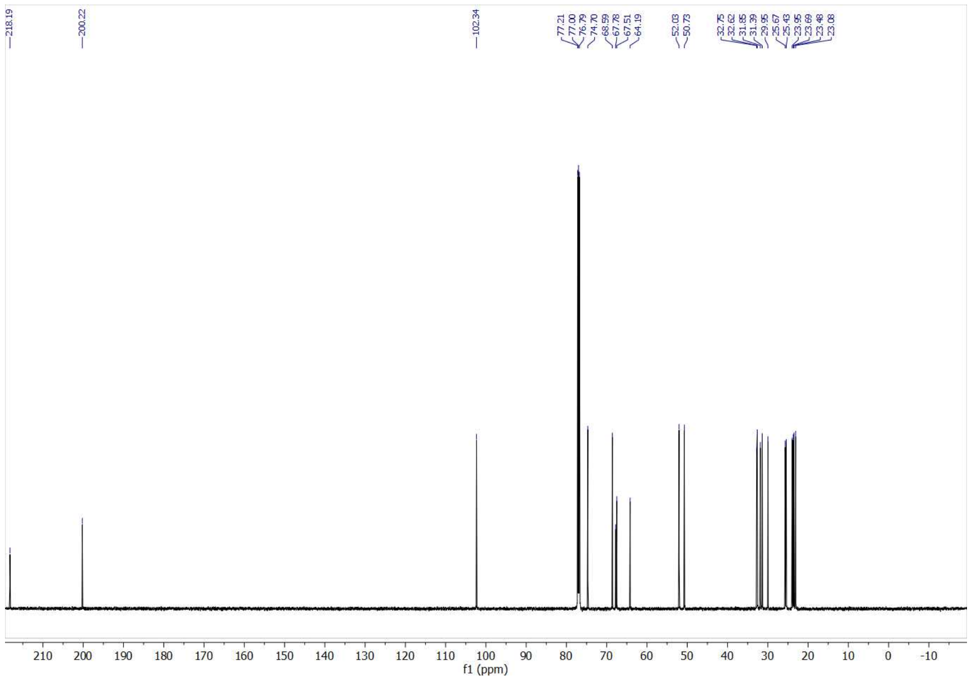

Fig. S15. ^{13}C NMR of the cycloadduct **7c**.

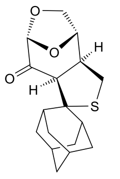

exo-**7d**

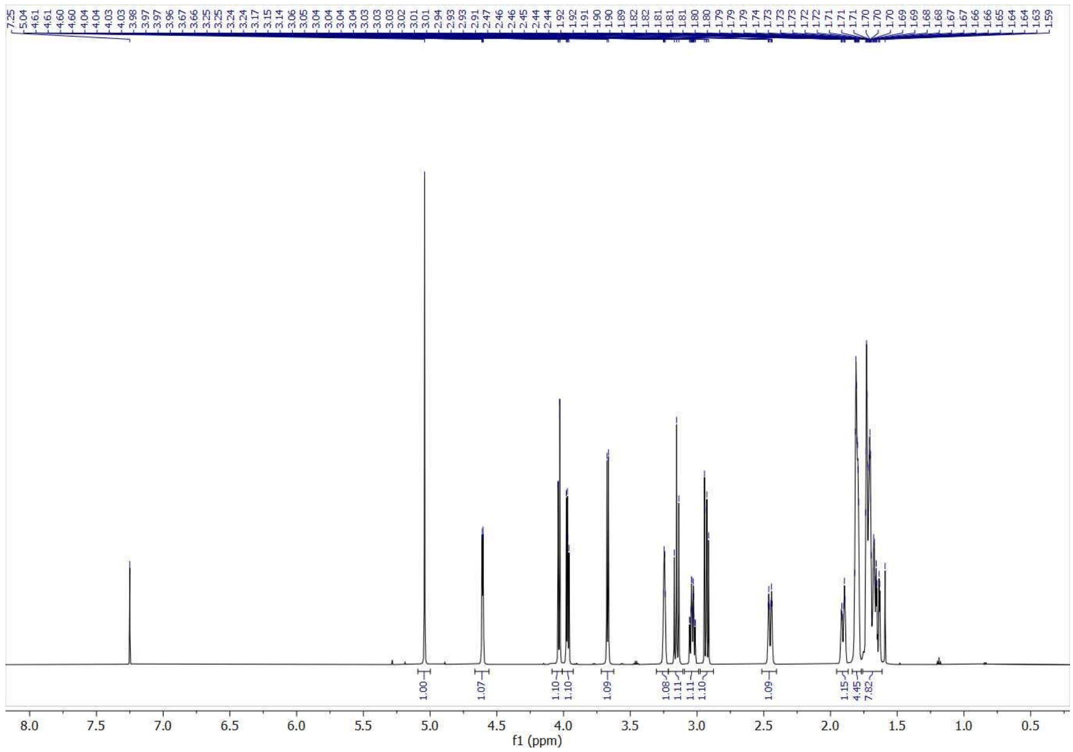

Fig. S16. ^{1}H NMR of the cycloadduct **7d**.

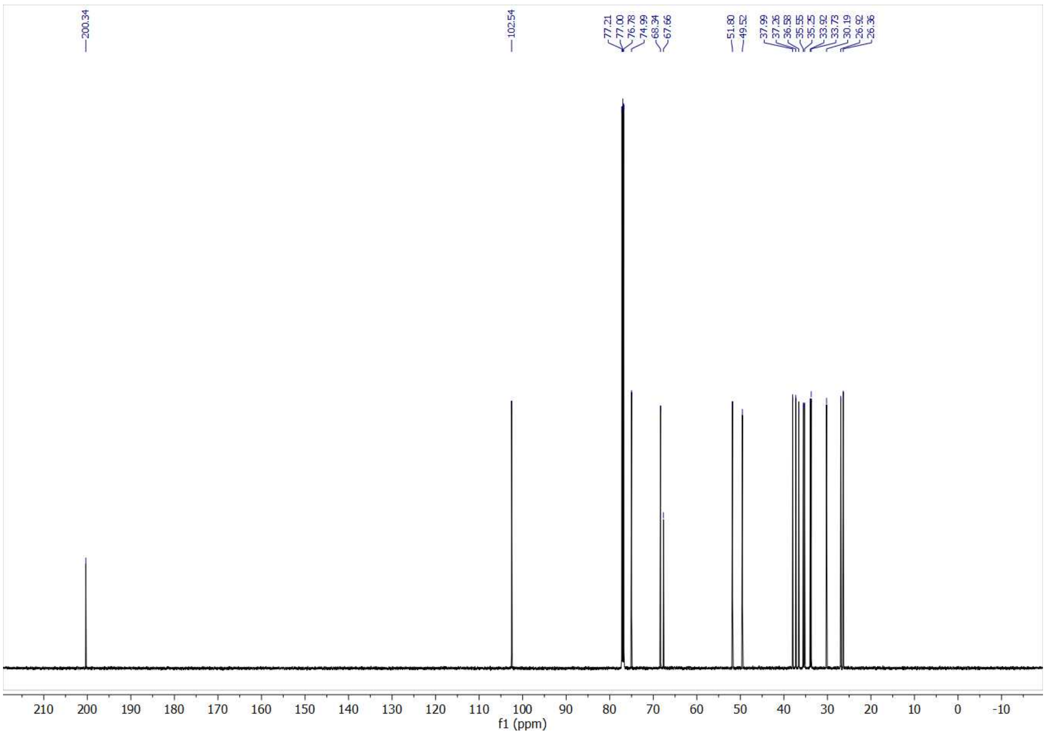

Fig. S17. ^{13}C NMR of the cycloadduct **7d**.

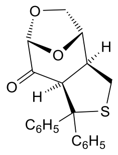

exo-**7e**

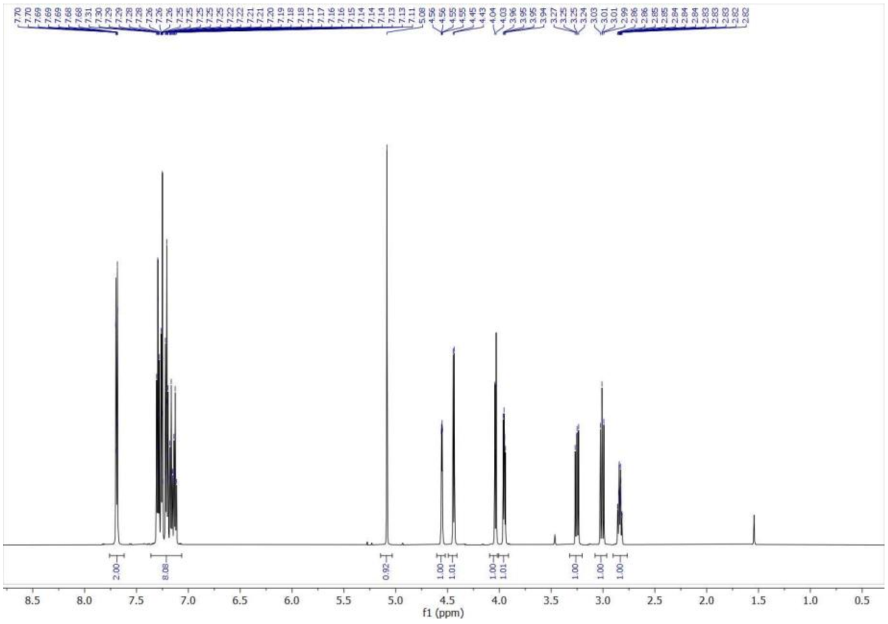

Fig. S18. ^{1}H NMR of the cycloadduct **7e**.

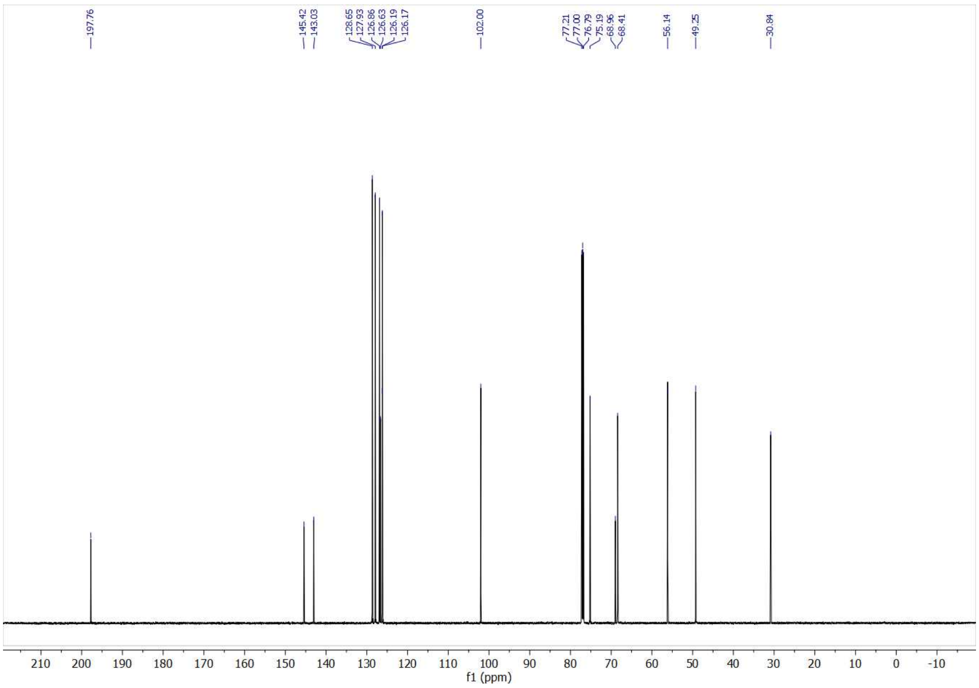

Fig. S19. ^{13}C NMR of the cycloadduct **7e**.

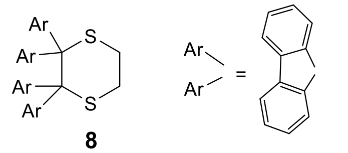

8

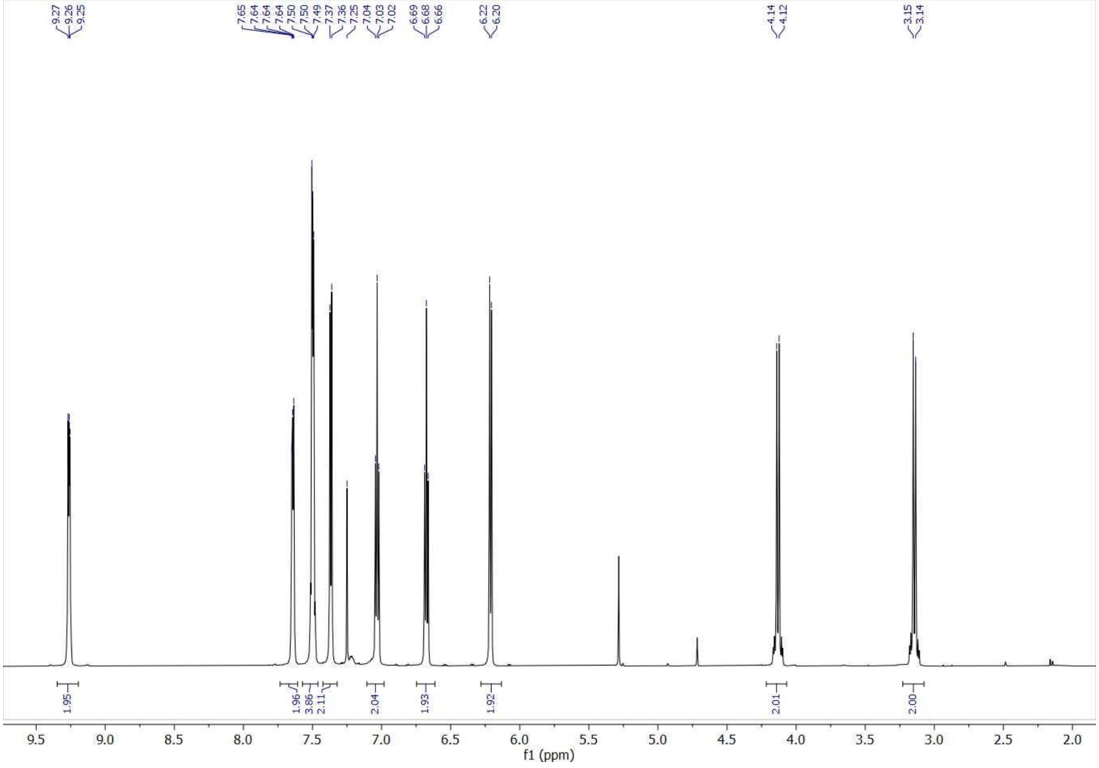

Fig. S20. ¹H NMR of the dimer **8**.

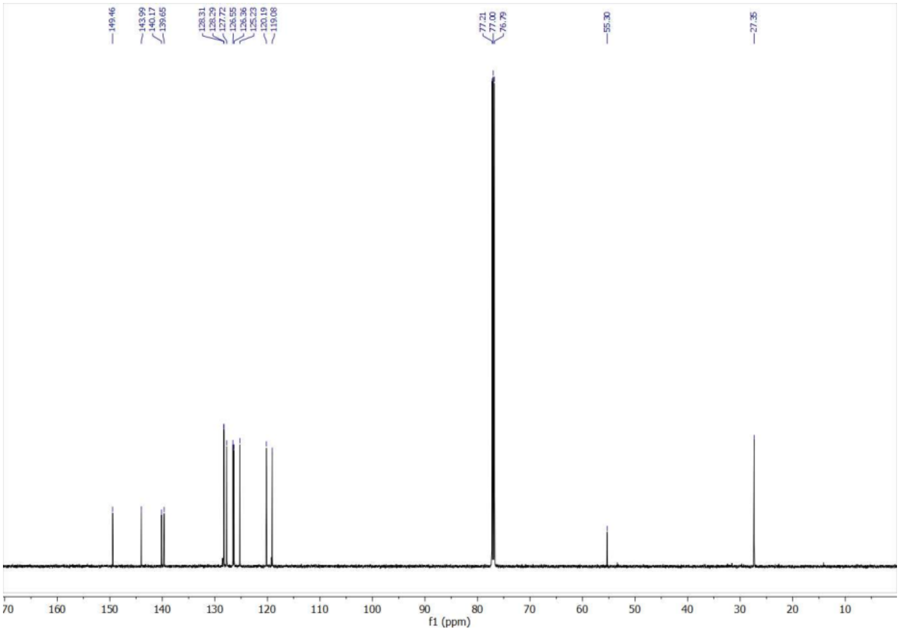

Fig. S21. ¹³C NMR of the dimer **8**.

3. Crystal Structure Determinations of Cycloadducts *exo*-7a, *exo*-7b, and *exo*-7d

Experimental:

X-ray diffraction data for *exo*-**7b** and *exo*-**7d** were collected on an XtaLAB Synergy, Dualflex, HyPix diffractometer. In case of *exo*-**7a** on a XtaLAB Synergy, Dualflex, Pilatus 300K diffractometer. Integration of the intensities and corrections for Lorentz effects, polarization effects, and analytical absorption were performed with CrysAlis PRO [S6] Using Olex2 [S7]; the structure was solved with the SHELXT [S8] structure solution program using Intrinsic Phasing and refined with the SHELXL [S9]. refinement package using Least Squares minimization. The hydrogen atoms were introduced in the calculated positions with an idealized geometry and constrained using a rigid body model with isotropic displacement parameters equal to 1.2 of the equivalent displacement parameters of their parent atoms. The molecular geometries were calculated by the PLATON program [S10]. The relevant crystallographic data are given in Table S1 (SI).

Atomic coordinates, displacement parameters, and structural factors of the analyzed crystal structures are deposited with the Cambridge Crystallographic Data Centre CCDC (reference number: 2368368 and 2368369 and 2083333) (Table S1) [S11].

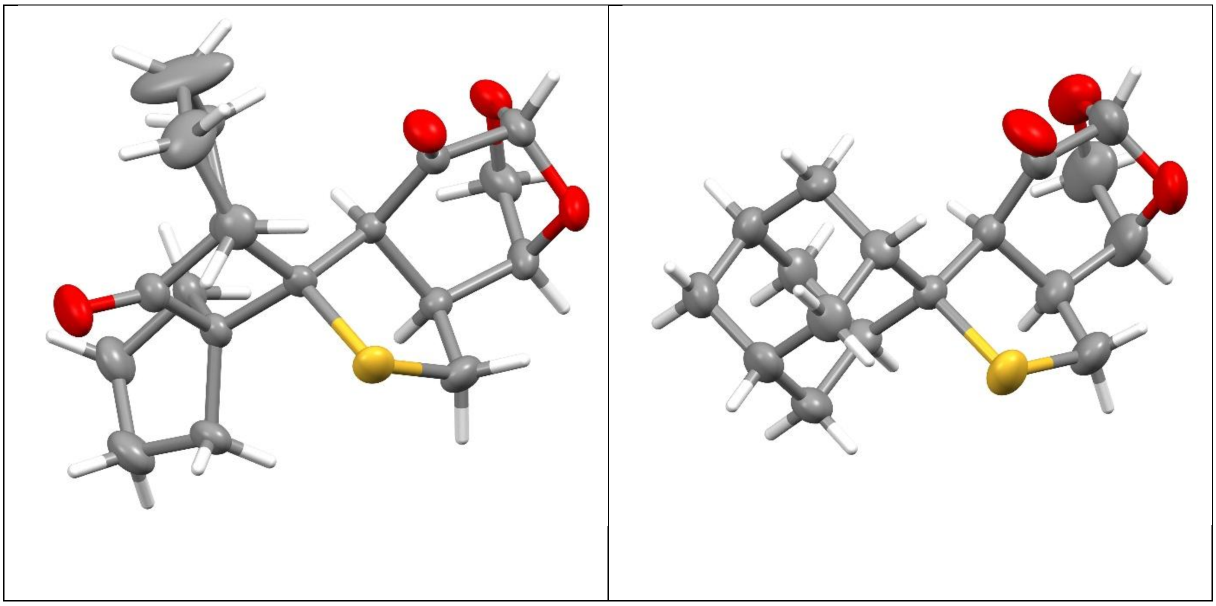

Fig. S19. A view of the molecular structure of compounds *exo*-7b (left) and *exo*-7d (right). Displacement ellipsoids are drawn at the 50% probability level. In the case of *exo*-7b only one of two molecules from independent unit cell is shown.

Table S1. Crystal data and structure refinement for *exo*-**7b**, *exo*-**7d**, and *exo*-**7a**.

Identification code	*exo*-**7b**	*exo*-**7d**	*exo*-**7a**
Empirical formula	$C_{17}H_{22}O_3S$	$C_{38}H_{48}O_8S_2$	$C_{15}H_{20}O_4S$
Formula weight	306.40	696.88	296.37
Temperature/K	293(2)	293(2)	100(1)
Crystal system	hexagonal	orthorhombic	orthorhombic
Space group	$P6_4$	$P2_12_12_1$	$P2_12_12_1$
a/Å	13.42560(10)	9.79260(10)	8.98979(4)
b/Å	13.42560(10)	18.21830(10)	12.29437(9)
c/Å	14.26360(10)	19.4673(2)	12.87559(6)
α/°	90	90	90

β/°	90	90	90
γ/°	120	90	90
Volume/Å^3	2226.52(4)	3473.05(5)	1423.060(14)
Z	6	4	4
ρ_{calc}mg/mm^3	1.371	1.333	1.383
μ/mm^{-1}	2.001	1.822	2.122
F(000)	984.0	1488.0	632.0
Crystal size/mm^3	0.62 × 0.42 × 0.16	0.35 × 0.28 × 0.08	0.59 × 0.31 × 0.15
2Θ range for data collection	7.604 to 153.254°	6.644 to 153.15°	
Index ranges	-14 ≤ h ≤ 16, -16 ≤ k ≤ 16, -15 ≤ l ≤ 17	-12 ≤ h ≤ 10, -22 ≤ k ≤ 22, -23 ≤ l ≤ 23	-11 ≤ h ≤ 11, -15 ≤ k ≤ 13, -15 ≤ l ≤ 16
Reflections collected	27143	33175	56411
Independent reflections	2848[R(int) = 0.0279]	7024[R(int) = 0.0429]	2999 [R_{int} = 0.0343, R_{sigma} = 0.0115]
Data/restraints/parameters	2848/1/191	7024/0/434	2999/0/186
Goodness-of-fit on F^2	1.037	1.055	1.080
Final R indexes [I>=2σ (I)]	R_1 = 0.0239, wR_2 = 0.0599	R_1 = 0.0361, wR_2 = 0.0964	R_1 = 0.0238, wR_2 = 0.0639
Final R indexes [all data]	R_1 = 0.0243, wR_2 = 0.0601	R_1 = 0.0376, wR_2 = 0.0975	R_1 = 0.0239, wR_2 = 0.0639
Largest diff. peak/hole / e Å^{-3}	0.15/-0.13	0.26/-0.23	0.29/-0.18
Flack parameter	0.000(5)	0.000(5)	0.000(3)
CCDC number	2368368	2368369	2083333

4. References

[S1] Klepp, J.; Dillon, W.; Lin, Y.; Feng, P.; Greatrex, B. W. Preparation of (–) Levoglucosenone from cellulose using sulfuric acid in polyethylene glycol, *Org. Synth.* **2020**, *97*, 38–53.
DOI: 10.15227/orgsyn.097.0038.

[S2] Huisgen R.; Penelle, J.; Mlostoń, G.; Padias, A. B.; Hall, H. K. *J. Am. Chem. Soc.* **1992**, *114*, 266–274.
DOI: 10.1021/ja00027a035.

[S3] Mlostoń, G.; Celeda, M.; Palusiak, M.; Heimgartner, H.; Denel-Bobrowska, M.; Olejniczak, A. B. Ambident reactivity of enolizable 5-mercapto-1*H*-tetrazoles in trapping reactions with *in situ* generated thiocarbonyl *S*-methanides derived from sterically crowded cycloaliphatic thioketones. *Beilstein J. Org. Chem*. **2025**, *21*, 1508−1519.
DOI: 10.3762/bjoc.21.113.

[S4] Mlostoń, G.; Huisgen, R. Acid–base reactions of adamantanethione *S*-methylide and its spiro-1,3,4-thiadiazoline precursor. *Tetrahedron* **2001**, *57*, 147–151.
DOI: 10.1016/S0040-4020(00)00988-1.

[S5] Huisgen, R.; Li, X.; Mloston, G.; Fulka, C. Reactions of Thiobenzophenone *S*-Methylide with Thiocarbonyl Compounds. *Eur. J. Org. Chem*. **2000**, 1695–1702.
DOI: 10.1002/(SICI)1099-0690(200005)2000:9<1695::AID-EJOC1695>3.0.CO;2-4.

[S6] CrysAlisPRO software system, Oxford Diffraction/Agilent Technologies UK Ltd, Yarnton, England, 2015.

[S7] Dolomanov, O. V.; Bourhis, L. J.; Gildea, R. J.; Howard, J. A. K.; Puschmann, H. OLEX2: A Complete Structure Solution, Refinement and Analysis Program. *J. Appl. Crystallogr*. **2009**, *42*, 339–341.
DOI: org/10.1107/S0021889808042726.

[S8] Sheldrick, G. M. SHELXT-Integrated space-group and crystal-structure determination. *Acta Cryst. Sect. A*: Foundations and Advances **2015**, *71*, 3–8.
DOI: org/10.1107/S2053273314026370.

[S9] Sheldrick, G. M. Crystal structure refinement with SHELXL. *Acta Crystallogr. Sect. C: Struct. Chem*. **2015**, *71*, 3–8.
DOI: org/10.1107/S2053229614024218.

[S10] Spek, A. L. Structure validation in chemical crystallography. *Acta Crystallogr. Sect. D: Biol. Crystallogr.* **2009**, *65*, 148–155.
DOI: org/10.1107/S090744490804362X.

[S11] Groom, C. R.; Bruno, I. J.; Lightfoot, M. P.; Ward, S. C. The Cambridge Structural Database, *Acta Cryst.* **2016**, *B72*, 171–179.
DOI: org/10.1107/S2052520616003954.
